# The use of genome-scale metabolic network reconstruction to predict fluxes and equilibrium composition of N-fixing versus C-fixing cells in a diazotrophic cyanobacterium, *Trichodesmium erythraeum*

**DOI:** 10.1186/s12918-016-0383-z

**Published:** 2017-01-19

**Authors:** Joseph J. Gardner, Nanette R. Boyle

**Affiliations:** Department of Chemical and Biological Engineering, Colorado School of Mines, Golden, CO 80401 USA

**Keywords:** Flux balance analysis, Constraints based modeling, Flux variability analysis, Nitrogen cycle, Diazotrophy, Marine cyanobacteria

## Abstract

**Background:**

Computational, genome based predictions of organism phenotypes has enhanced the ability to investigate the biological phenomena that help organisms survive and respond to their environments. In this study, we have created the first genome-scale metabolic network reconstruction of the nitrogen fixing cyanobacterium *T. erythraeum* and used genome-scale modeling approaches to investigate carbon and nitrogen fluxes as well as growth and equilibrium population composition.

**Results:**

We created a genome-scale reconstruction of *T. erythraeum* with 971 reactions, 986 metabolites, and 647 unique genes. We then used data from previous studies as well as our own laboratory data to establish a biomass equation and two distinct submodels that correspond to the two cell types formed by *T. erythraeum*. We then use flux balance analysis and flux variability analysis to generate predictions for how metabolism is distributed to account for the unique productivity of *T. erythraeum*. Finally, we used *in situ* data to constrain the model, infer time dependent population compositions and metabolite production using dynamic Flux Balance Analysis. We find that our model predicts equilibrium compositions similar to laboratory measurements, approximately 15.5% diazotrophs for our model versus 10-20% diazotrophs reported in literature. We also found that equilibrium was the most efficient mode of growth and that equilibrium was stoichiometrically mediated. Moreover, the model predicts that nitrogen leakage is an essential condition of optimality for *T. erythraeum*; cells leak approximately 29.4% total fixed nitrogen when growing at the optimal growth rate, which agrees with values observed *in situ.*

**Conclusion:**

The genome-metabolic network reconstruction allows us to use constraints based modeling approaches to predict growth and optimal cellular composition in *T. erythraeum* colonies. Our predictions match both *in situ* and laboratory data, indicating that stoichiometry of metabolic reactions plays a large role in the differentiation and composition of different cell types. In order to realize the full potential of the model, advance modeling techniques which account for interactions between colonies, the environment and surrounding species need to be developed.

**Electronic supplementary material:**

The online version of this article (doi:10.1186/s12918-016-0383-z) contains supplementary material, which is available to authorized users.

## Background

Nitrogen serves a critical role in the metabolism of all organisms. As a key component in nucleic acids and proteins, it is required for healthy growth and it is often one of the most limiting nutrients for optimal yield. Human intervention via the Haber-Bosch process for the production of ammonia has greatly shifted the global nitrogen cycle, however many ecosystems still rely heavily on biological nitrogen fixation. One such ecosystem is in the open ocean, which is a nutrient-limited environment and organisms that thrive here have evolved to thrive in deplete conditions. *Trichodesmium* is a genus of filamentous diazotrophic (nitrogen fixing) cyanobacteria that not only flourishes in this environment but provides bio-available nitrogen for surrounding species. *Trichodesmium* is responsible for fixing roughly 100 TgNy^-1^ of nitrogen annually (42% of global N fixation) [1] and has been reported to ‘leak’ 30-50% of the nitrogen it fixes [2]. The genus is ubiquitous in marine environments; it is found in environments as diverse as the Mediterranean Sea [3], the Pacific Ocean [4–6], and the Great Barrier Reef where it has implications not only as a source of nitrogen, but also as a center for eutrophication [7]. It dwells primarily near the surface [8] and can swell to occupy acres of the ocean or sea. Despite its prominence in the global nitrogen cycle, most research efforts have focused on *in situ* sampling and therefore little has been done to model and or predict the effect of different environmental factors on the growth and nitrogen fixation rates in *Trichodesmium.*

*Trichodesmium* is a colonial cyanobacteria which grows in multicellular filaments called trichomes, each containing about 130 cells [9]. *Trichodesmium* is a non-heterocystous cyanobacterium which means it does not employ specialized cells (heterocysts) for nitrogen fixation. Instead, nitrogen fixation and photosynthesis can occur within the same cell. Most non-hetrocystous cyanobacteria separate oxygen producing photosynthesis from nitrogenase by using temporal separation; they fix nitrogen at night when the cellular metabolism is in respiration mode (consuming carbohydrates stored during the day by photosynthesis). *Trichodesmium* is unique in its mechanism to fix nitrogen, it fixes nitrogen during the day while simultaneously fixing carbon via photosynthesis. Respiration rates in *Trichodesmium* are reported to be higher than other cyanobacteria, which ensures a micro- or anaerobic environment and thus minimizes the potential poisoning of nitrogenase by oxygen [10, 11]. Nitrogenase is only expressed in a subset (10-20%) of cells consecutively arranged in the middle of the trichome. These diazotrophic cells only express photosystem I because photosystem II produces oxygen [10, 12–15]. Current characterization of *Trichodesmium* is limited predominantly to population level observations due to its genetic intractability and difficulty to culture. While several laboratory studies investigating the complex genome [16–18], transcriptome [19, 20], and proteome [21] have been published, most relate to populations level or sparse *in situ* studies in diverse, non-ideal growth conditions. A handful of other recent studies report on the morphology/structure of the cells [8, 10, 22, 23] and how cells respond to iron, nickel, and other nutrient stresses [24–27]. Despite the availability of these studies, they are limited in scope and do not provide a complete picture of *Trichodesmium* on a cellular scale. The long doubling time (57-98 h), low growth density (~100mg/L) [24, 28–30], and lack of genetic tools have limited laboratory based research on *Trichodesmium*, especially compared to other diazotrophic cyanobacteria such as *Anabaena* and *Cyanothece*.

This work presents the first genome-scale reconstruction of a colony forming diazotrophic cyanobacterium, *T. erythraeum*, a leader in marine nitrogen fixation. It models biological optimization of metabolic exchange and biomass creation through Flux Balance Analysis (FBA) and Flux Variability Analysis (FVA) [31] to predict the different metabolic behaviors of the two cell types formed by *T. erythraeum*, nitrogen fixing cells and photoautotrophic cells, constrained by laboratory or published data/observations. The models described in this work illustrate how *T. erythraeum* divides labor between two cells stoichiometrically and applies the first step towards a multi-objective framework of these bilaterally operating cells. These results are extended to understand overall population compositions and metabolite production rates to visualize what role metabolite passage plays in formation of these complex colonies via dynamic Flux Balance Analysis (dFBA) [32] and a population co-optimization algorithm. This model lays the foundation for future colonial cyanobacteria characterization and integration with *in situ* and transcriptomic data for *T. erythraeum*.

## Results

### Biomass composition and growth rates

**Fig. 1:**
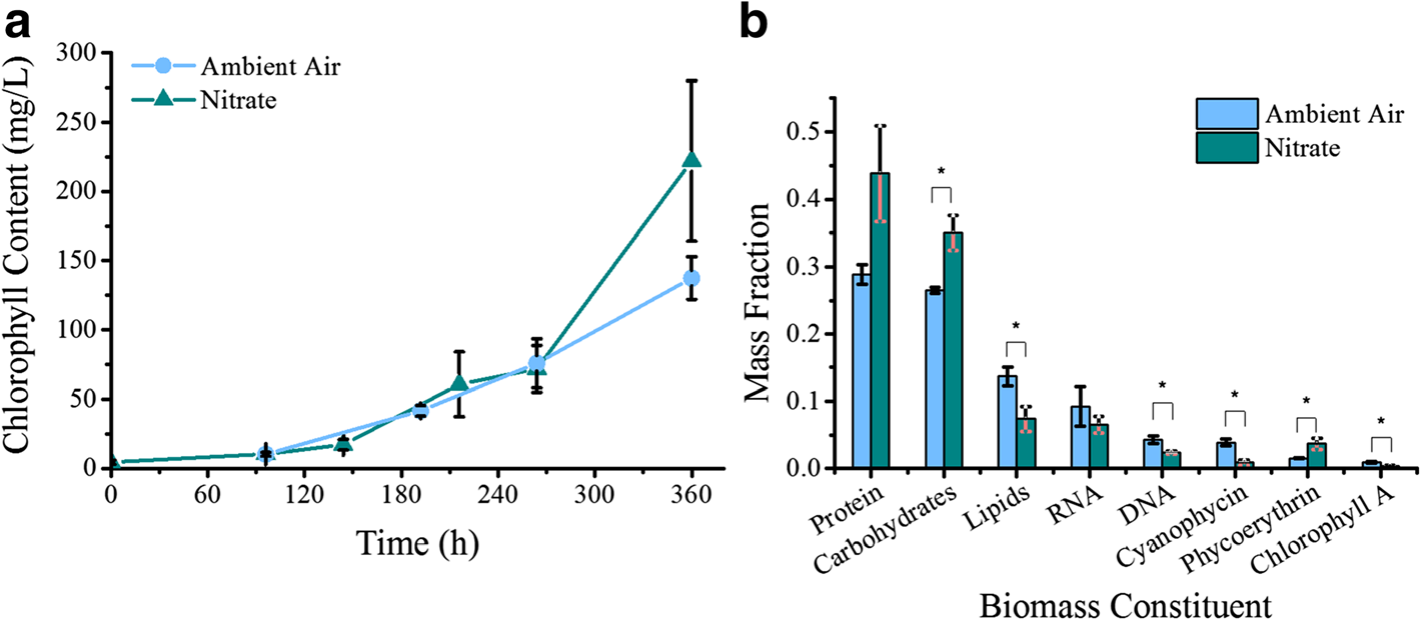
Growth curves and biomass compositions of *T. erythraeum.* Cells were grown with either ambient air (circles/left and blue) or potassium nitrate (triangles/right and dark cyan) as the nitrogen source in YBCII medium and growth was monitored by measuring total chlorophyll a content. **a**) Growth curves of cells in different nitrogen sources and computational growth. Error bars represent standard deviation from 3 biological replicates. **b**) Biomass composition of *T. erythraeum*. The major elements of biomass were measured directly from cultures grown on diatomic nitrogen (ambient air) or potassium nitrate. Error bars represent standard error from 6 biological replicates

Cells were grown in YBC-II medium in ambient air (79% N_2_) or supplemented with either 100 *μ* mol KNO_3_ as a nitrogen source. Growth was monitored by measuring total chlorophyll content (see Fig. 1a). These data were then used to calculate the growth rate and doubling time assuming exponential growth (Table 1).

**Table 1:** Growth rates and doubling times of *T. erythraeum*

Nitrogen source	Growth rate (d^-1^)	Doubling time (h)
Ambient Air	0.0108 ± 5.14 × 10^-4^	64.4 ± 5.10
KNO_3_	0.0120 ± 5.65 × 10^-4^	58.1 ± 2.86

Reported **e**rror is standard error $\left(\frac{\sigma }{\sqrt{n}}\right)$ where *n* = 3 biological replicates

The biomass composition of *T. erythraeum* was measured in order to formulate an accurate biomass formation equation. The composition of cells grown on both ambient air and nitrate were measured (see Table 2 as described in the methods section. The “soluble pool” is a collection of soluble metabolites that are well conserved between organisms for survival and includes small sugars, energy carrying molecules, and other small molecules. To accurately represent proteins and lipids, the amino acid and fatty acid composition of cells were measured as well (see Additional file 8: Tables S1 and S2). We attempted to grow cells on ammonium because we hypothesized that this would remove the necessity of diazotroph formation and enable all the cells in the trichome to be carbon-fixing. This would allow biomass measurement of photoautotrophs directly; however, growth on ammonium in the laboratory was not possible as seems to be consistent with some other cyanobacteria. Therefore we used the composition of cells grown on N_2_ as the average biomass composition for all subsequent modeling efforts.

**Table 2:** Biomass composition of *T. erythraeum*

Metabolite	Mass fraction (g/g DW)		Biomass coefficient (mmol/g DW)	
	N_2_	KNO_3_	N_2_	KNO_3_
Protein	0.289	0.438	2.12 × 10^-4^	2.66 × 10^-4^
Phycoerythrin^a^	1.54 × 10^-2^	3.67 × 10^-2^	2.64 × 10^-2^	4.46 × 10^-4^
Cyanophycin^a^	3.80 × 10^-2^	9.33 × 10^-2^	6.96 × 10^-2^	4.31 × 10^-2^
Carbohydrate	0.265	0.351	4.59 × 10^-1^	5.33 × 10^-1^
RNA	9.18 × 10^-2^	6.51 × 10^-2^	2.88 × 10^-3^	2.46 × 10^-3^
DNA	4.28 × 10^-2^	2.40 × 10^-2^	1.39 × 10^-3^	1.09 × 10^-3^
Lipids	0.1370	7.40 × 10^-2^	3.89 × 10^-3^	3.00 × 10^-3^
Phycocyanin^b^	2.60 × 10^-2^	3.67 × 10^-2^	4.45 × 10^-2^	5.37 × 10^-2^
Chlorophyll^b^	8.91 × 10^-3^	0.424 × 10^-3^	9.99 × 10^-3^	7.37 × 10^-3^
Soluble Pool	2.86 × 10^-2^	2.86 × 10^-2^	3.79 × 10^-2^	3.79 × 10^-2^
Total	0.914	1.04	-	

The biomass equation is the molar concentration of the metabolite predicted by the computational molar mass and uses the values from the ambient air (N_2_) grown cultures. The “Soluble Pool” is a collection of soluble metabolites that are more or less conserved between organisms for survival (including small sugars, energy carrying molecules, etc.) ^a^Subset of protein measurement. ^b^Subset of lipid measurement

### Network reconstruction and manual curation

Reconstructing a complete genome-scale metabolic network from a genomic sequence required several iterations. The first draft of the network was created using an automated genome-scale model algorithm, the SEED RAST [33] and contained 956 reactions. Automated metabolic network reconstructions rely on homology to well characterized and annotated model organisms such as *E. coli* or *S. cerevisiae*. Therefore, the unique metabolic pathways for photosynthesis and nitrogen fixation were not accounted for in the initial draft. The initial automated draft had several gaps and missing reactions, therefore several iterations of manual curation were necessary to fill in missing reactions and infer the presence of reactions that were not predicted based on homology to model organisms. First, we focused on closing the balance on reactions associated with biomass and cellular energy pathways (light harvesting, ATP cycling, and Redox reactions); this was done by referencing metabolic pathway databases such as BioCyc [34] and KEGG [35]. We then compared our draft network to genome scale reconstructions of other related organisms, including *Cyanothece* ATCC 51142 [36], *Synechocystis* sp. PCC 6803 [37], *Synechococcus* sp. PCC 7002 [38], *Arabidopsis thaliana* [39], *Phaeodactylum tricornutum* [40], and *Chlamydomonas reinhardtii* [41, 42]*.* Through comparison, we added more photosynthesis-specific metabolic reactions which were predicted to be present in the genome based on BLAST results. Finally, transport reactions included in the model were selected based on proteomic data or diffusion (CO_2_, H_2_O, N_2,_ etc.) [16]. Manual curation efforts built the model out to a maximum of 1035 reactions; closer inspection of the reactions revealed that several were predicted by the SEED algorithm but had no significant homology to the *T. erythraeum* genome and were non-essential, therefore they were removed. The current draft of the genome-scale metabolic model (Additional file 1: Table S1 and Additional file 2: Table S2) for *T. erythraeum* contains 971 reactions: 1 biomass formation equation (based on experimental data), 9 macromolecule synthesis and condensation reactions, 27 exchange reactions, 38 transport reactions (validated by proteomics data), and 907 metabolic reactions. These reactions involve 647 unique genes and 986 metabolites. Despite our manual curation efforts, the model still has 215 dead end reactions: 113 are involved in lipid, amino acid, or pyrimidine/purine metabolism and are bypassed by summary reactions (see Additional file 3 for a complete list). Manual curation of the model led us to identify 9 genes which are present in the genome based on homology but not annotated, 5 genes encoding enzymes with related functions we assume to be promiscuous and 1 gene which is required for the production of biomass but was not present in the genome based on homology (see Table 3).

**Table 3:** Unannotated metabolic reactions in the *T. erythraeum* genome but included in the model based on homology to related organisms and/or to close gaps for biomass formation

Pathway	Proposed function	E.C. Number	Gene	Annotated function	Closest organism
Newly/Improved Annotated					
Amino Acid Metabolism	L-alanine: glyoxylate aminotransferase	2.6.1.44	Tery_3167	Serine: glyoxylate transaminase	*Leptolyngbya* sp. NIES 3755
	L-serine: pyruvate aminotransferase	2.6.1.44, 2.6.1.45, 2.6.1.51	Tery_3167	Serine: glyoxylate transaminase	*Leptolyngbya* sp. NIES 3755
	L-aspartase	4.3.1.1	Tery_1328	Fumarase	*Nitrosococcus oceani*
	L-arogenate: 2-oxoglutarate aminotransferase	2.6.1.79	Tery_0293	L-aspartate aminotransferase	*Pleurocapsa* sp. PCC 7327
	L-threonine ammonium-lyase	4.3.1.19	Tery_4742	Pyridoxal-5’-phosphate-dependent enzyme, beta subunit/cysteine synthase A	*Zymomonas mobilis* subsp. NRRL B-12526
Isoprenoid Synthesis	Tocopherol phytyltransferase	2.5.1.117	Tery_3881	Homogentisate phytyltransferase	*Nostoc* sp. NIES-3756
Pigment Metabolism	Chlorophyllide-a: NADP^+^ oxidoreductase	1.1.5.31.3.1.75	18445Tery_3563	NmrA-like	*Amborella trichopoda*
Secondary Carbon Metabolism	Citramalate synthase	2.3.1.182	Tery_2253	2-Isopropylmalate synthase	*Dehalococcoides mccartyi VS*
Sulfur Metabolism	O-succinylhomoserine thiol lyase	2.5.1.48	Tery_0352	8-Amino-7-oxononanoate synthase	*Nostoc* sp. PCC 7524
Tricarboxylic Acid Cycle	Isocitrate lyase	4.1.3.1	Tery_4268	2,3-Dimethylmalate lyase/methylisocitrate lyase	*Candidatus Nitrososphaera gargensis*
Assumed Promiscuous					
Amino Acid Metabolism	4-Hydroxyglutamate transaminase	2.6.1.23	Tery_0293	L-aspartate aminotransferase	*T. erythraeum*
Cofactor and Energy Carrier Metabolism	Dihydroneopterin *P*_*i*_ dephosphorylase	3.6.1.1	Tery_1519	Inorganic diphosphatase	
	Dihydroneopterin *PPP*_*i*_ dephosphorylase	3.6.1.1	Tery_1519	Inorganic diphosphatase	
Lipid Metabolism/Secondary Carbon Metabolism	Glycoaldehyde dehydrogenase	1.2.1.21	Tery_2599	Aldehyde dehydrogenase	
Nucleotide Metabolism	3’-5’-Nucleotide phosphodiesterase: cAMP	3.1.4.17	Many	3’-5’-Nucleotide phosphodiesterase: NMP	
Missing (but Essential) Gene					
Cofactor and Energy Carrier Metabolism	(R)-Pantoate:NADP^+^2-oxidoreductase	1.1.1.169	None	None	None

### Flux balance analysis

A *T. erythraeum* trichome is made up of cells with two distinct metabolic modes: photoautotrophic and diazotrophic. Each cell type was modeled separately and thus required a different set of constraints to define the cell type. The specific constraints applied to each cell type based on literature and experimental data are provided in the methods section (Table 6). Growth associated ATP demand is assumed to be identical to *Cyanothece* sp. ATCC 51142 [36]: 544 mmol ATP (g DW h)^-1^. Maintenance energy, represented by the reaction EN_ATP: ATP + H_2_O → ADP + H^+^ + Pi was adjusted until the predicted growth rate matched published experimental growth rates (0.0146 h^-1^) [29]. Maintenance energy demands were found to be: 64.3 mmol ATP (g DW h)^-1^ for photoautotrophs and 67.2 mmol ATP (g DW h)^-1^ for diazotrophs. We hypothesize that this number is significantly higher than heterotrophic bacteria for two reasons: (i) maintaining a micro-aerobic or anaerobic environment in the cells for nitrogenase is energetically demanding and (ii) we do not constrain the photon absorption beyond the amount provided to the cells in the laboratory despite knowledge that cells aren’t 100% efficient at light harvesting. The COBRA toolbox [43] was used to evaluate the biomass yields for each cell type separately subject to published growth rates, carbon dioxide uptake, and nitrogenase activity (see Table 4). An .m file to run the model in Matlab with appropriate constraints has been included in the supplemental files (Additional file 4).

**Table 4:** Predicted yields and selected fluxes for T. erythraeum

Cell type	Carbon Uptake (moles C/ g DW)	NH_4_^+^ Flux (mole NH_4_^+^/ mole C)	Biomass yield (g DW/mole C)	Biomass yield (g DW/mole N)
Diazotroph	0.0572	0.204	17.5	55.3
Photoautotroph	0.0643	-0.0996	15.6	156

Biomass and exchange differences between the two cell types are a result of different sources of energy. The carbon mass percent (45.8%) is identical for both cell types because the same biomass formation equation was used (based on growth on N_2_)

**Fig. 2:**
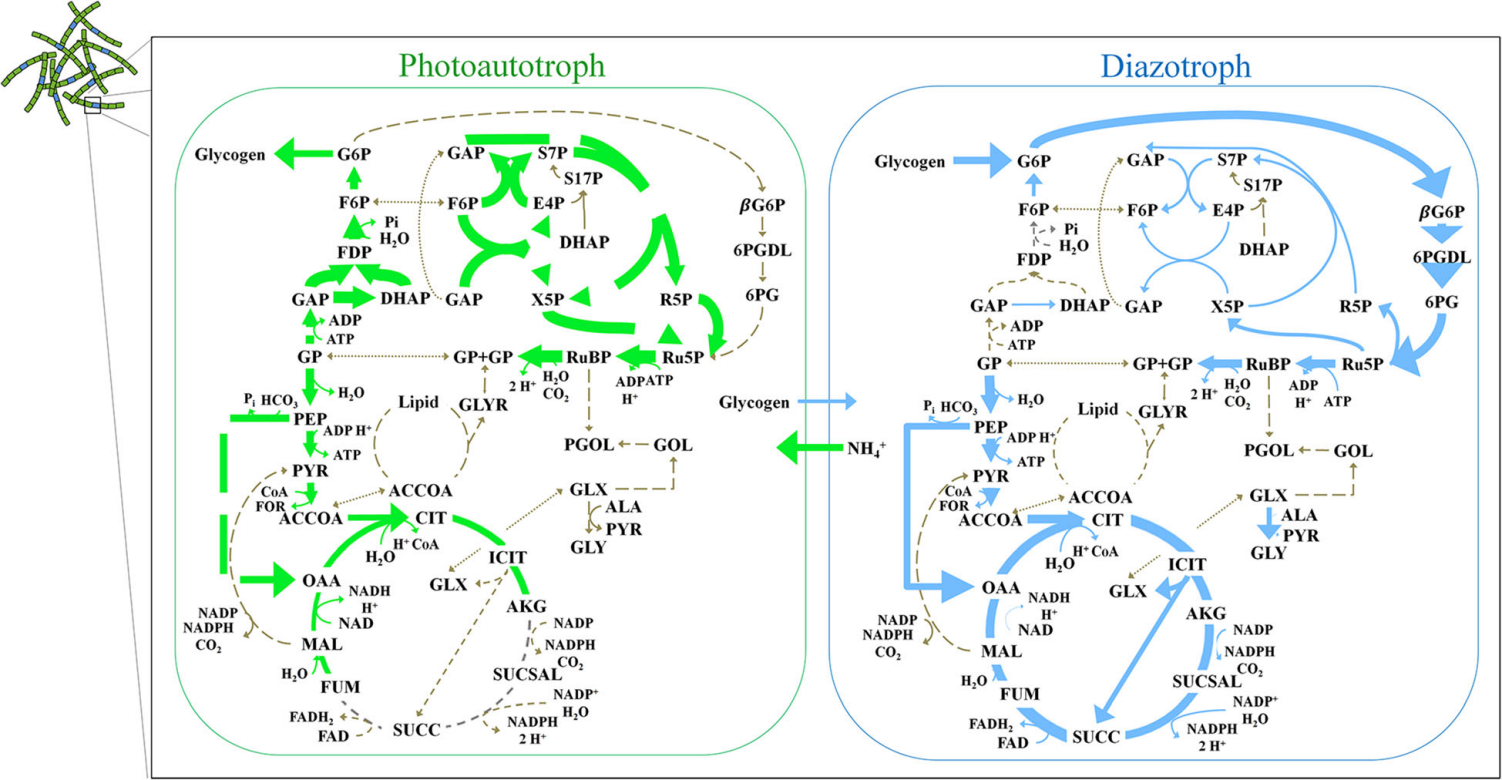
Predicted central metabolic fluxes for (**a**) diazotrophic and (**b**) photoautotrophic cells of T. erythraeum. Flux balance analysis was used to predict fluxes for both metabolic modes in a trichome of *T. erythraeum*. The thickness of the arrows depicts the amount of flux through the reaction normalized to the uptake of the carbon source. Dotted gray lines are available unused pathways. Diazotrophic cells have high flux through respiratory pathways, this protects nitrogenase from oxygen. As expected, photoautotrophic cells have high fluxes in the Calvin Benson Basham Cycle, which is the carbon fixing pathway. Abbreviations for metabolites are provided in abbreviations section. Full catalog of fluxes are provided in Additional file 5: Table S5

Flux maps were generated in order to visualize carbon trafficking within the cell for both cell types (illustrated in Fig. 2 with data in Additional file 5: Table S5) subject to the constraints specified in Table 6. As expected, the model for a photoautotrophic cell exhibited high flux through the non-oxidative pentose phosphate pathway and gluconeogenesis. Moreover, the highest recorded flux is through ribulose-1,5-bisphosphate carboxylase/oxygenase (RuBisCO), the enzyme responsible for carbon fixation. The TCA and glyoxylate cycles are partially inactive; the majority of energy is produced through lower glycolysis or photosynthesis and the TCA Cycle’s main utility is in precursor biosynthesis. The diazotroph, on the other hand, has higher flux in the pathways associated with respiration: the oxidative pentose phosphate pathway and TCA cycle in particular displayed substantial activity. The glyoxylate shunt also has high flux, indicating crucial differences in how metabolism is regulated in the different cell types.

Another utility of a FBA model is the ability to predict essential genes. We performed an *in silico* gene knockout analysis and identified 275 genes as essential in phototrophic cells and 253 in diazotrophic cells (see red-coded genes in Additional file 6: Genes Table S6). Essential genes are frequently linked to biomass relevant compound synthesis (like pigments and amino acids), carbon and/or nitrogen fixation, or glycolysis. Genes and reactions which decrease growth rate but were not lethal were frequently linked to central carbon processing. These gene knockout results are corroborated by reaction analysis, where reactions are removed from the model instead of genes; this analysis found 370 reactions essential in a photoautotroph and 363 in a diazotroph (see red coded reactions in Additional file 6: Reactions Table S6). Most reactions overlapped, but 6 carbon fixation reactions and 3 gluconeogenesis reactions were unique to essentiality in photoautotrophs while ammonium output and 2 nitrogen fixation reactions were unique to diazotrophs. Unfortunately, *T. erythraeum* has not been reported to be genetically tractable and therefore it is not possible to experimentally validate these results.

### Flux variability analysis (FVA)

**Fig. 3:**
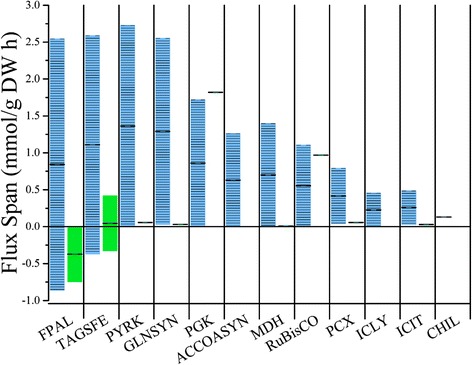
Allowable variation in flux for central metabolic reactions. Bars visualize the flux variability through important pathways. . Blue/lined bars are diazotrophic fluxes and green/solid bars are photoautotrophic fluxes. High variability implies adaptable responses while low variability implies a narrow essentiality for biomass and energy generation. Greater variability was displayed between cell types in similar pathways including glycolysis (FPAL, PYRK, PGK), the Calvin Cycle (TAGSFE, RuBisCO) and nitrogen processing (GLNSYN) while smaller variability was through the TCA cycle (ACCOASYN, MDH, ICIT) and the glyoxylate shunt (ICLY). Non-zero fluxes were found for the photoautotroph in PGK and RuBisCO while reversibility was found in both cell types for FPAL and TAGSFE. Greater variabilities through carbon processing were due to redundancies in cellular processing, while less variability or non-zero variability was found in non-redundant pathways like carbon and nitrogen fixation. Abbreviations are found under “Enzymes” in the Abbreviations section

Flux balance analysis uses optimization techniques to predict fluxes in the cell, but most (if not all) genome-scale models are underdetermined so there exists the possibility of multiple flux distributions that lead to the same solution (in our case biomass/growth rate). To evaluate the flexibility in our model, we performed flux variability analysis (FVA) to estimate how much variability a particular reaction can tolerate and still give the same solution. Enzymes, selected from important pathways and by comparing different metabolic distributions in the diazotrophic and photoautotrophic FBA, are shown in Fig. 3 and a table of bounds provided as Additional file 7: Table S7. Overall, photoautotrophic cells display tighter metabolic regulation and have required flux through every major pathway except for nitrogen fixation. In comparison, the diazotrophic model displays high variability through these central pathways with the exception of nitrogenase function. For the photoautotrophic model, two genes exhibit fully nonzero flux: phosphoglycerate kinase (PGK) and ribulose-1,5-bisphosphate carboxylase/oxygenase (RuBisCO). Otherwise, two genes, D-fructose-1,6-bisphosphate D-glyceraldehyde 3-phosphate lyase (FPAL) in upper glycolysis, and sedoheptulose-7-phosphate: D-glyceraldehyde-3-phosphate (TAGSFE) in the pentose phosphate pathway exhibit wide ranges with TAGSFE possibly functioning in both directions while the same optimum is achieved. The complete tricarboxylic acid (TCA) cycle is invariably nonfunctional for energy metabolism in optimization of the photoautotrophs (ACCOASYN, MDH, ICLY, and ICIT) and only functions for precursor biosynthesis. Diazotrophs ultimately demonstrate more variability due to their energy source as more redundancy exists in carbon oxidation than in photosynthesis: all reactions can accommodate zero flux except nitrogen fixation. FPAL and TAGSFE both show reversibility (same as photoautotroph with respect to TAGSFE). Although there are similar patterns in the different cell types, energy metabolism is significantly different.

### Dynamic flux balance analysis

**Fig. 4:**
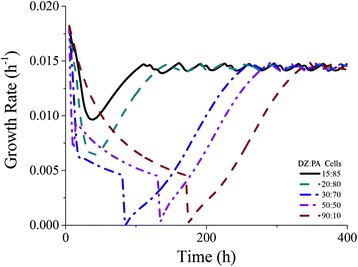
Growth rate evolution at different initial compositions. The growth rate between time points, taken by measuring biomass generated by dFBA at t_i_ = t and t_f_ = t + 5, was plotted versus time. Each line represents a different initial composition as listed on the graph (15:85 corresponds to 0.1 diazotroph:0.9 photoautotroph, etc.). All other conditions were held equal, and carbon uptake and nitrogen fixation were adapted from physiological data over 400 h intervals. The gray line represents the initial composition being set to the equilibrium composition. Growth was unachievable at initial compositions of 0:100 and 100:0

**Fig. 5:**
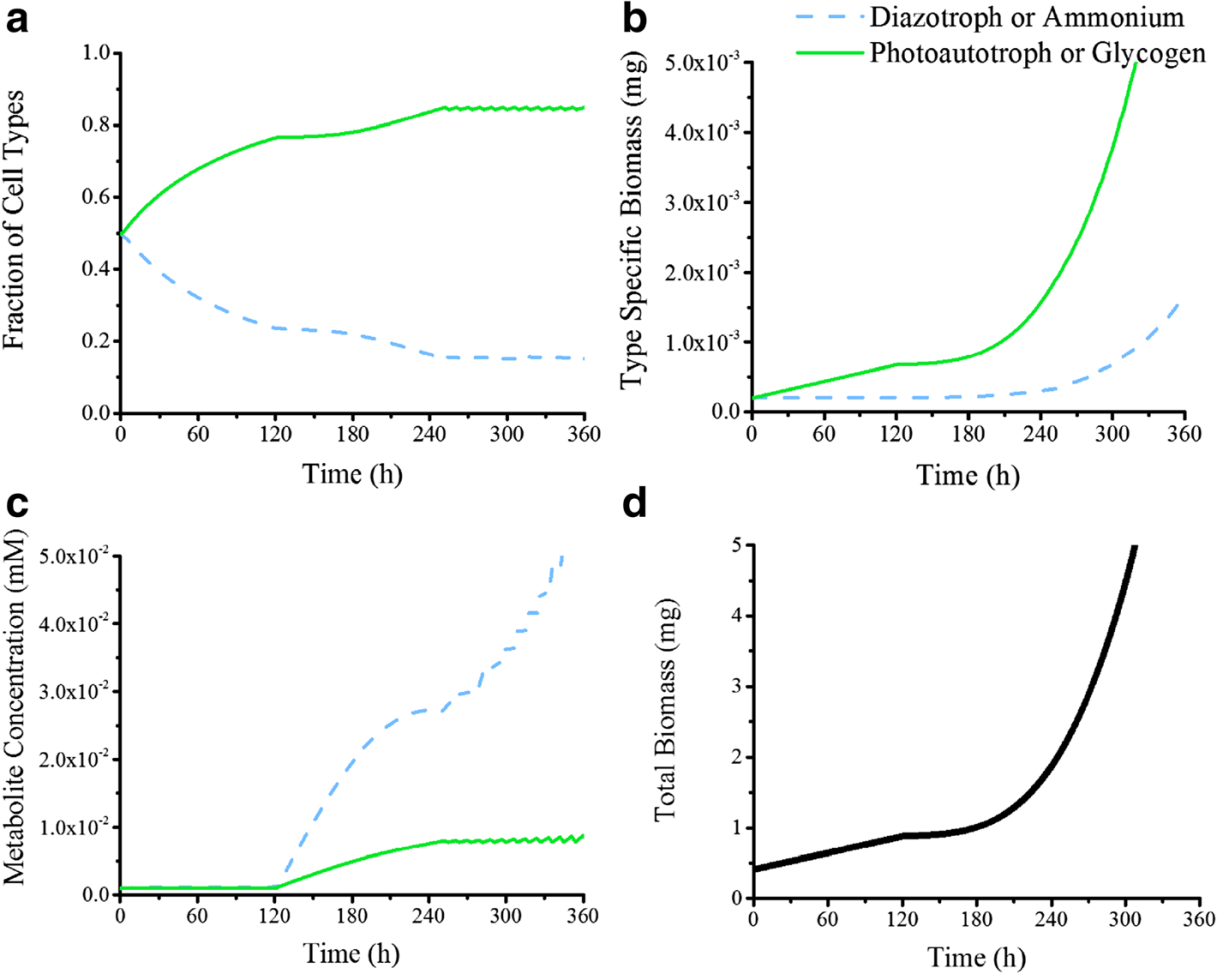
Computational population rates for *T. erythraeum*. Dotted blue lines indicate diazotroph or fixed nitrogen, green lines indicate photoautotroph or glycogen. **a** Fraction of population for each cell type. Three phases of growth are present: linear redistribution of cells to create enough photoautotrophs, steady preferential allocation to photoautotrophs to drive biomass generation, and achievement of equilibrium. Equilibrium is 0.1544 diazotroph and 0.8456 photoautotroph. **b** Growth rates of each cell type. Biomass is modeled using a batch reactor model with growth rate determined by FBA using the genome-scale reconstruction and time steps of 1 h. **c** Medium concentration for metabolites. These indicate the metabolite accumulation in the medium as determined by the amount of metabolite produced by each cell type less the metabolite consumed plus the amount of metabolite already existing in solution. **d** Total growth rate of population. This is the total growth rate and is plotted with experimental growth rates in Fig. 1. It is calculated by adding the two biomasses in *B* together

Dynamic Flux Balance Analysis (dFBA) was conducted to predict how well the model performed when compared to laboratory experiments using the same data to constraint the model as reported earlier (see Table 6). No additional constraints on diatomic nitrogen or carbon dioxide uptake were applied except for the demand from the other cell type We used the dFBA model to test a number of different growth conditions and hypotheses. First, to predict the equilibrium trichome composition, the model was run with an inoculum of equal parts photoautotrophic and diazotrophic cells. Once equilibrium was determined, initial concentrations of each cell type were calculated from experimental data and the simulation was re-run to validate that the most efficient growth and metabolite production occurred at this equilibrium. The resulting colony fractions to which the cells invariably converged were found to be 0.15:0.85 diazotrophs:photoautotrophs, similar to experimental evidence [10]. To understand how the initial inoculum effects the lag phase of the cells, we ran simulations for a number of different inoculums. Given that the algorithm requires a sequential progression of metabolism (with the photoautotroph calculating its metabolism and then the diazotroph reacting), a “grace period” of 3 time steps was built in where the cells could borrow substrates from an arbitrary cache in their environment so that they could grow initially. However, if a population is unable to produce metabolites or biomass after those three consecutive time steps, the cells were assumed to be unviable and were terminated. How the growth rate evolves over time to reach the experimental value (0.0146 h^-1^) is shown in Fig. 4. The expected growth rate is obtained rapidly for near-equilibrium starting populations (15:85, 20:80) and more slowly for populations far from equilibrium (90:10). Populations of 0:100 and 100:0 were nonviable. More detailed figures of how the model predicts composition changes when far from equilibrium is shown in Fig. 5 with initial equality of cell types. Cells appear to have three distinct growth phases, the first where they are adjusting their ratios to move closer toward equilibrium (lag phase) while not able to leak metabolites. When the cells are near equilibrium (at ~0.22 diazotroph: 0.78 photoautotroph), they are able to grow exponentially, but are unable to leak ammonium consistent with their exponential growth (Fig. 5c). When they finally reach equilibrium, growth and ammonium leakage is exponential, indicating most efficient growth. In the dFBA model, photoautotrophic cells are unable to grow without diazotrophic cells and vice versa; however, if the models are optimized assuming nutrient unlimited environments (unrestricted availability of glycogen or ammonium), the photoautotroph can grow at a rate of 0.0182 h^-1^ and the diazotroph can grow at a maximum rate of 0.0188 h^-1^. From these results, the equilibrium found in nature and this study represents the best-case growth scenario for *T. erythraeum*; this growth appears to be stoichiometrically and metabolically motivated instead of regulated through other means.

**Fig. 6:**
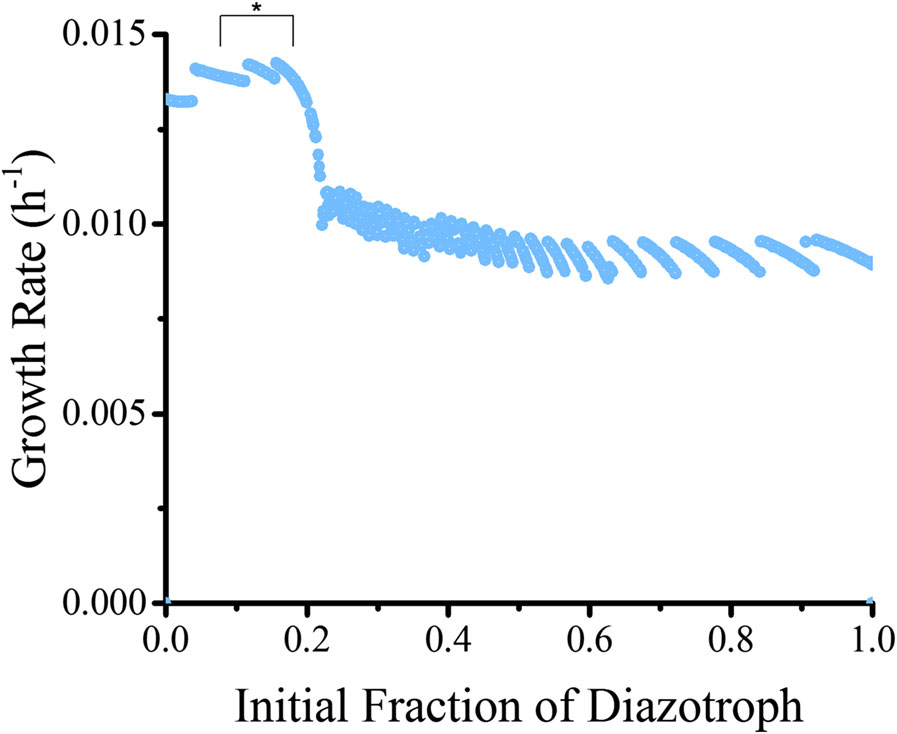
Growth rate at different initial compositions. The average growth rate dependent on different relative values of diazotroph to photoautotrophic cells. 1000 iterations were generated with random values of initial biomass for each cell type. The initial fraction was calculated using the ratio of these two randomly generated numbers and dFBA was run for 400 h to simulate population development over that time period. Where growth rate was zero over three or more time steps (1 h each) or the cells were unable to manufacture their own nutrients, cell death was assumed and occurred at zero for initial fraction of diazotroph. Optimal growth was found at 0.1544 and suboptimal non-zero growth was found with diazotroph dominated initial populations. The bracket and asterisk refers to the literature predicted equilibrium concentration of cells

**Fig. 7:**
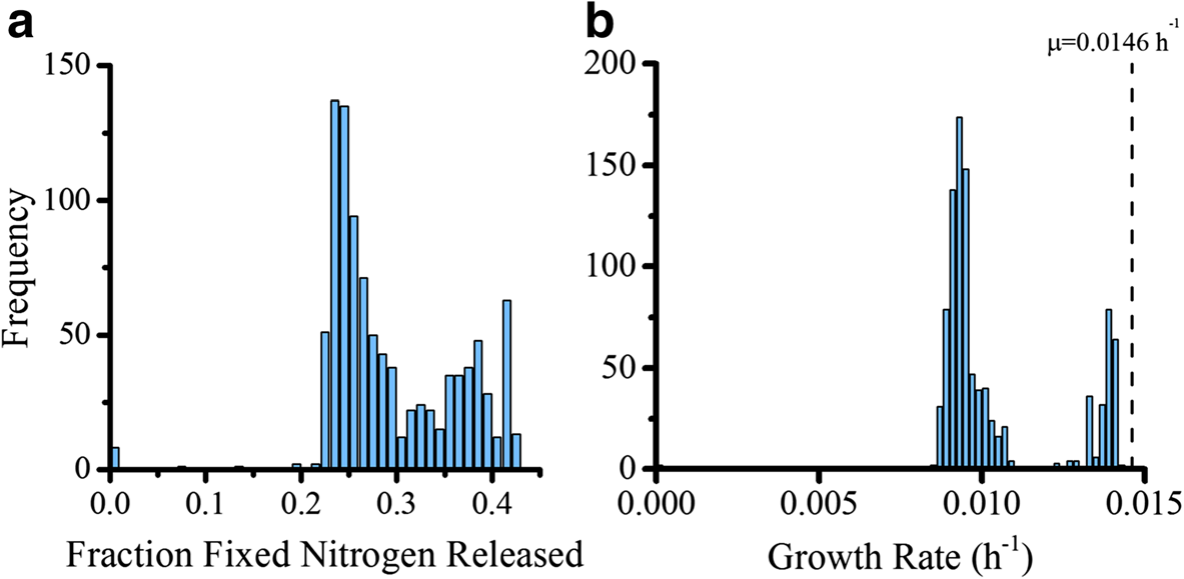
Nitrogen and biomass production based on variable initial cell concentrations. 1000 iterations were run with randomly generated initial biomasses of each cell type and run for 400 h to simulate laboratory measured behavior. **a** Fraction of fixed nitrogen released calculated by the average amount of fixed nitrogen accumulated in the medium of a Batch Reactor model divided by the average fixation rate over the time period. Values are clustered between 25% and 45% except for non-growth cases where no nitrogen was released because death was assumed. **b** Growth Rate over all iterations. Non-growth cases resulted in zero, but otherwise a bimodal distribution existed. This can be coupled with 0.1544:0.8456 diazotroph: photoautotroph ratio determined by simulation.photoautotroph ratio determined by simulation. The dotted black line refers to laboratory measured growth at 0.0146 h^-1^

This experiment generated several important parameters to compare against literature. Intercellular metabolite production, cell fraction, and percent released of total fixed nitrogen were all predicted using dFBA. The model was run for 1000 iterations with different inoculum compositions to investigate the effect of initial compositions of population development. The generated values for an ideal population (beginning with equilibrium concentrations of each cell type) are summarized in Table 5. The model over predicts nitrogenase flux (0.490 mol N_2_ (g DW h)^-1^ for the model versus 0.132 mol N_2_ (g DW h)^-1^ for published measurements but is similar to literature values for carbon dioxide exchange (0.922 mol CO_2_ (g DW h)^-1^ for the model versus 0.927 mol CO_2_ (g DW h)^-1^ for laboratory results) [29]. The model resulted in a total biomass of 13.8 mg/L when simulated at equilibrium starting composition, compared to the 10–40 mg/L found in our laboratory experiments. Variability in growth occurred as illustrated in Fig. 6: growth rates peaked at 0.0146 h^-1^ for growth rate as expected due to constraints (47.5 h for doubling time) at initial cell fractions of 0.15 diazotrophs: 0.85 photoautotrophs). Initial diazotroph concentrations at zero resulted in no growth while photoautotroph dominated inoculums had the minimum nonzero values of 2.80⨯10^-4^ h^-1^. Fixed nitrogen release rate distributions and growth rate distributions through all iterations are illustrated in Fig. 7a and b respectively.

**Table 5:** Productivities of *T. erythraeum* according to literature, laboratory experiments, and the dFBA model

Source	Population fraction (Diazotroph: Photoautotroph)	Growth rate (h^-1^)	Doubling Time (h)	% Nitrogen released	Nitrogenase flux (mmol N (g DW h)^-1-^)	CO_2_ Uptake (mmol CO_2_ (g DW h)^-1^)
Literature	0.1:0.9 – 0.2:0.8 [10]	0.0146 [29]	47.5 [29]	7.7 [62] – 52 [22]	0.132 [29]	0.927 [29]
Boyle Lab	N/A	0.0108 ± 8.53 × 10^-4^	64.4 ± 5.10	N/A	N/A	N/A
dFBA Model	0.1544: 0.8456	0.0146	47.5	39.7%	0.490	0.922

N_E_ corresponds to exported nitrogen in the form of ammonium

**Fig. 8:**
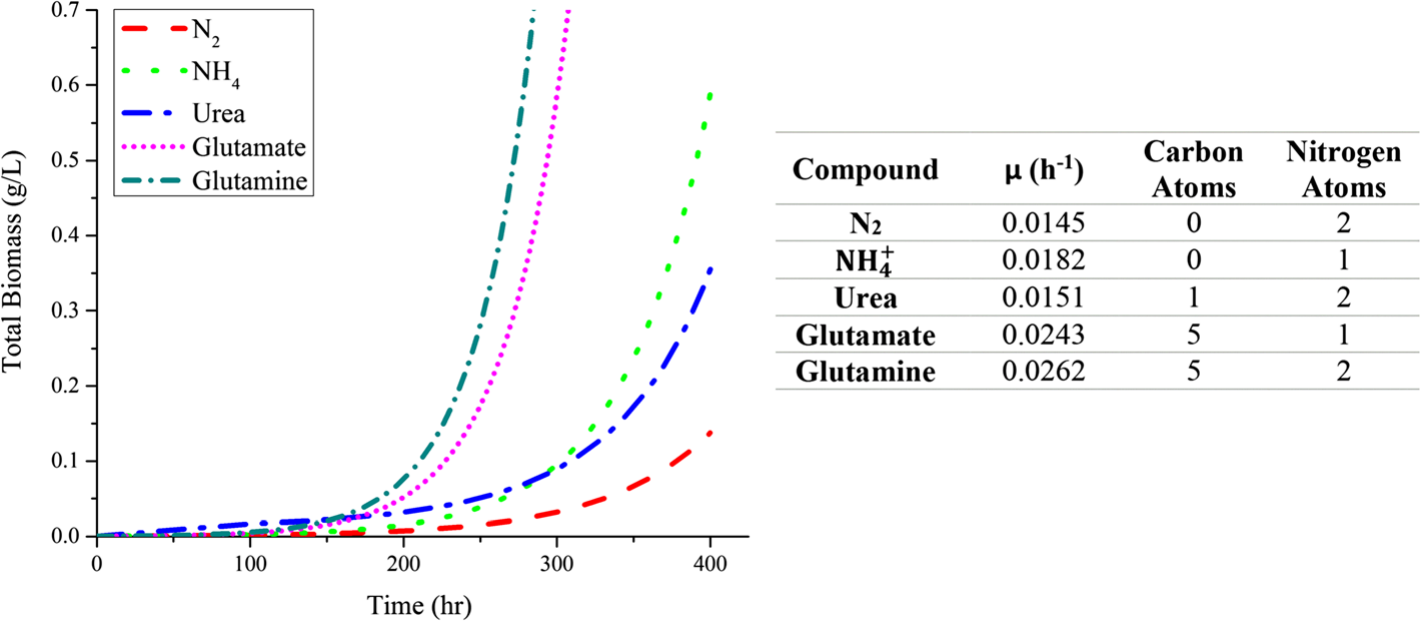
Predicted *in silico* growth rates on different nitrogen and nitrogen/carbon sources. Each line represents an excess of a compound. The slowest rate is given by ambient nitrogen which is the same set of values used for the computational curve. The accompanying table relays growth rates and nitrogens or carbons in the source

Finally, dFBA was conducted to investigate the effect of different nitrogen sources on the growth rate of *T. erythraeum*. All other constraints on the mode were held constant and the resulting growth curves are given in Fig. 8. As expected, growth rates increase with increasing levels of nitrogen reduction and carbon in the nitrogen source. Unfortunately, growth on nitrogen sources other than N_2_ or NO_3_ have proven to be problematic and there is no evidence of *T. erythraeum*’s ability to utilize other nitrogen sources.

## Discussion

In this work, we present a genome-scale metabolic network reconstruction of *T. erythraeum* which has been experimentally validated and used to predict growth under a variety of conditions.

### Experimental data

An important aspect of any metabolic model is to collect experimental data to aid in the development of the model (biomass equation) and for validation (metabolic production and cellular equilibrium). Major biomass constituents were measured experimentally with direction from literature to define the scope of the biomass constituents (such as inclusion of biliproteins and cyanophycin [8, 23]). It should be noted that *T. erythraeum* differs from other nitrogen fixing organisms and cyanobacteria. Species like *Cyanothece* and *Anabaena* either temporally regulate nitrogen fixation using circadian rhythms or through spatial segregation by forming special cells called heterocysts. *T. erythraeum*, while it does exhibit some circadian regulation [44], can fix nitrogen at all times, day or night. It does form two cell types, but the only evidence of different structures is indicated by accumulation of starches [23]. Cyanophycin granules exist, but are distributed throughout the cells, indicating that they are nitrogen storage compounds rather than structural devices. After assessing the literature based behavior of *T. erythraeum* and its differences from other bacteria, biomass was evaluated when grown on nitrogen or nitrate. The existence of two separate cell types (diazotrophic and photoautotrophic) within a trichome implies a locally nitrogen limited and nitrogen replete environment. We sought to mimic these effects. As expected, the nitrogen provided to the cell culture has a significant impact on biomass composition, and cells partition their carbon in different ways depending on availability of reduced nitrogen. Since the reduction of diatomic nitrogen to ammonia requires a large amount of energy (16 ATP), it is likely that the cells activate their nitrogen sparing mechanisms in order to limit the amount of nitrogen needed for growth. This is evident in the ratio between carbohydrates and lipids (Fig. 1a), which is significantly lower – only 54% – in cells grown on diatomic nitrogen compared to those grown on nitrate. This is similar to reports of algae which accumulate lipids in response to nitrogen starvation [45–50]. Here, we assume that growth on diatomic nitrogen causes the cells to act like nitrogen is limiting. Cells grown on nitrate have an increased protein content, again highlighting that the cell is no longer acting as if nitrogen is limiting. This increase in protein is also commensurate with an increase in the major photosynthetic pigment-protein complex phycoerythrin. Cultures grown on diatomic nitrogen convert their fixed nitrogen to higher quantities of cyanophycin, a molecule used for nitrogen storage. Meanwhile, this study also sought to characterize the laboratory growth rate of *T. erythraeum* grown on different sources of nitrogen. Unfortunately, *T. erythraeum* showed a reticence to grow on ammonium in the laboratory and has no evidence of a putative transporters for amino acids, commensurate with heuristic knowledge of other cyanobacteria, which limited this experimentation. Even so, growth rates are similar to previous laboratory measurements (0.0108 h^-1^ for the Boyle Lab and 0.0146 h^-1^ from [29]) but are dissimilar to *in situ* studies with values measured as low as 1.46 × 10^-4^ h^-1^ in the North Atlantic [22]. This is not surprising, it is well known that the open ocean is extremely nutrient deplete and thus a challenging growth environment. Also, our measurements of growth on different nitrogen sources do not indicate a statistically significant disparity in growth rate based on nitrogen source despite significant changes in cellular composition. As such, the data implies that nitrogen availability is not the major limiting factor in cellular growth. This is supported by literature which demonstrates that *T. erythraeum* is much more efficient in higher CO_2_ concentrations [51]. This means that the major effect of nitrogen source is on cellular composition and implies that carbon fixation is the growth limiting function of *T. erythraeum*.

### Metabolic network reconstruction

Equipped with a high-level biological understanding of the organism, a genome scale reconstruction was built using the annotated genome, enzyme databases, models of related organisms, and the laboratory biomass measurements. The first draft of the genome-scale metabolic network for *T. erythraeum* was created using the SEED RAST algorithm [33] followed by manual curation. Manual curation was conducted by adding all possible reactions predicted by databases, similar organisms, and gaps based on data in primary literature and publicly available databases [52]. This was pruned to the current model (986 metabolites, 647 unique genes and 971 reactions) by vetting each reaction through BLAST and essentiality. These numbers are comparable to metabolic network reconstructions of similar organisms (Additional file 8: Table S3). Reactions relating to central metabolism, including carbon fixation, glycolysis, the TCA cycle, photosynthesis and the pentose phosphate pathway, are conserved between *T. erythraeum* and the other cyanobacteria listed in Additional file 8: Table S3. Biomass equations were based on the *Cyanothece* ATCC 51142 model [36] for lipid and protein formation. The reactions were mass balanced to predict molar masses, and the biomass measurements were interpreted using these computational predictions. Finally, closure was obtained through proteomic assessment of transport reactions in conjunction with the metabolic network and biomass equations. Overall, the manually curated model of *T. erythraeum* is a strong summary of the majority of metabolic processes and relates well to previous models.

**Fig. 9:**
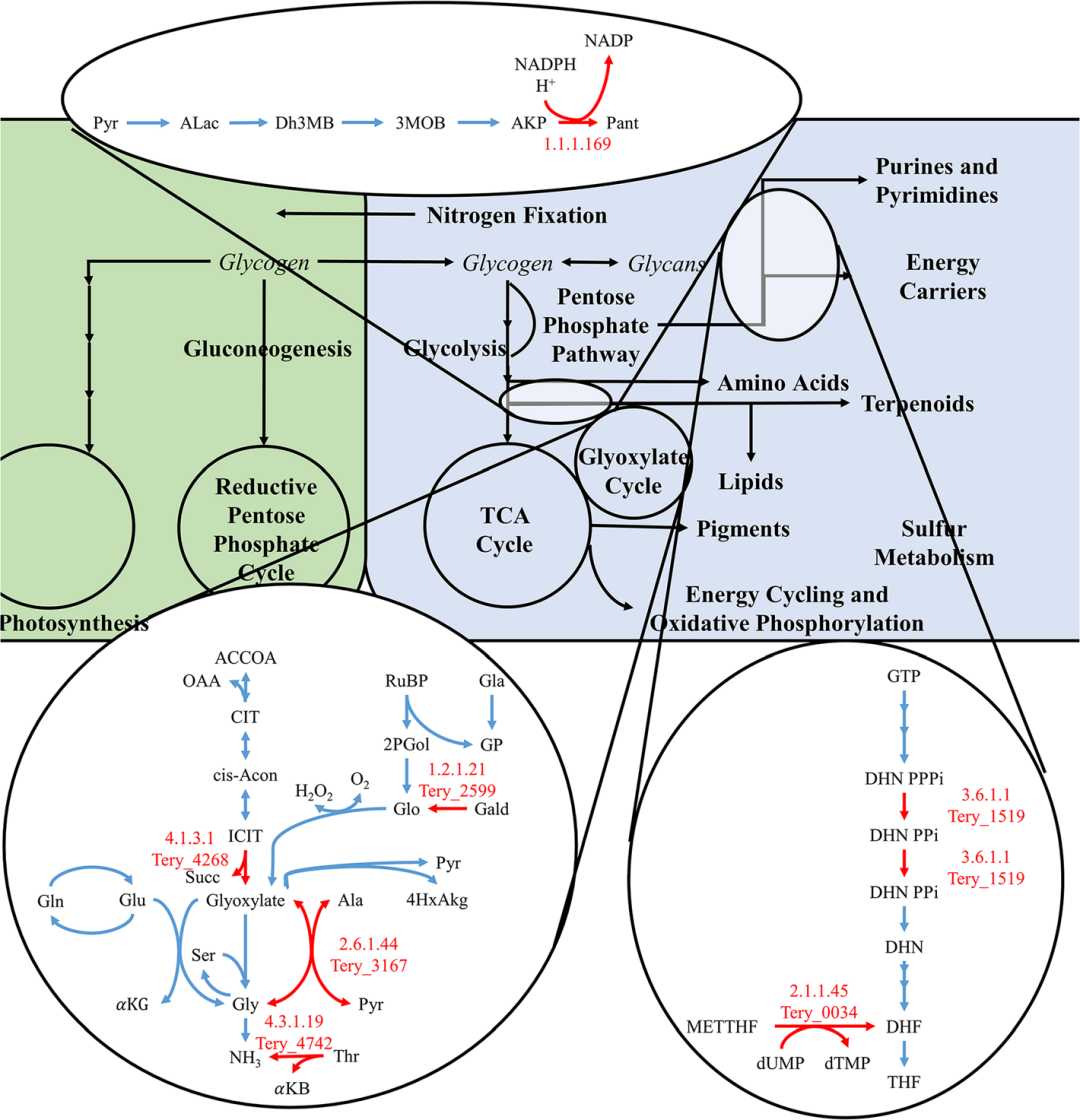
Genome scale reconstruction and filled pathways. The green cell is the photoautotroph, the blue cell is the diazotroph. Red arrows indicate missing pathways; appended genes indicate BLAST derived genes. Zoomed out sections are pathways that were completed. Omissions are summarized in Table 3 and describe amino acid metabolism and secondary carbon metabolism as the majority of gap-filled reactions. Only 1.1.1.169 (top zoom-out) had no significant correlation to another *T. erythraeum* or related organism enzyme

One of the more challenging aspects of building a metabolic network of a non-model organism is the lack of (or completeness of) genome annotation. However, in performing a genome-scale reconstruction, shortcomings in annotation can be identified. In order to achieve closure, 16 reactions were added that are not linked directly to a gene in the *T. erythraeum* genome by assuming enzyme promiscuity, hypothetical protein function, or a lack of evidence for its existence. To validate or refute the presence of these genes in the genome, we did extensive BLAST analysis with related organisms with more complete annotations (other cyanobacteria, *E. coli* and *A. thaliana*). We identified 5 genes encoding enzymes which had evidence of the desired function but were annotated differently (Table 3); we assumed these enzymes to be promiscuous and perform the necessary functions to fill gaps in the network. These assumptions were possible because the genes are often found to have redundant or generic function (the promiscuous genes include phosphatases and transaminases). We identified an additional 10 genes which were previously unannotated but are predicted to be present in the *T. erythraeum* based on homology to related organisms (Table 3). Most of these genes encode enzymes associated with the glyoxylate cycle, aminotransferases, and amino acid metabolism. Again, some of these enzymes have traditionally exhibited generic behavior (like transaminases), but other enzymes were unannotated due to their single-function synthetic purposes (like amino acid synthesis). Interestingly, enzymes associated with the glyoxylate cycle show the most similarity with ammonium oxidizing bacteria. The aminotransferases and amino acid metabolism enzymes, which generate some of the carbon molecules that participate in the glyoxylate cycle, show most similarity to other colony forming cyanobacteria like *Leptolyngbya* and *Nostoc*. This indicates that nitrogen metabolism may have impacts on peripheral carbon metabolism that merits future investigation. Finally, one enzyme which was necessary for the model to produce biomass, (R)-pantoate: NADP^+^ 2-oxidoreductase (E.C. 1.1.1.169), was not found to be present in the genome. This implies that *T. erythraeum* may have evolved an alternative pathway which has not yet been identified, or that the synthetic route for (R)-pantoate has significant structural differences. Main pathways included in the model along with the several of the newly annotated enzymes are presented in Fig. 9. Confidence levels in the model reflect the strength of prediction of the enzymes, with ones referring to the enzymes discussed above.

### Predicting central metabolic fluxes

With a vetted series of metabolic interactions, the model was used to study whole-cell behavior for both diazotrophs and photoautotrophs separately using flux balance analysis (FBA). The two cell types share the same genome but have distinct metabolisms based on differences in regulation due to cellular differentiation: diazotrophic cells provide biologically available nitrogen for the community and photoautotrophic cells provide a reduced carbon source, in the form of glycogen, to the diazotrophs. To model metabolic fluxes for each type of cell, we developed two sets of strict constraints (see Table 6) to reflect the laboratory constraints measured by literature [29]. We fit the predicted growth rate from the model by changing maintenance energy and used metabolite production (of the metabolites exchanged between cells) as the objective equation to ensure satisfaction of both objectives. As expected, flux distributions were specific to cell type. Carbon fixation is central to photoautotrophic function and is evident by high flux through the Calving Benson Bassham cycle. For diazotrophs, the absence of Photosystem II requires a functional TCA cycle for production of cellular energy. Interestingly, the oxidative pentose phosphate pathway was preferred over glycolysis, likely due to minimizing carbon oxidation and/or redox balancing. This same phenomenon explains the activation of the glyoxylate shunt which has evolved to conserve carbon [53–56]. The flux maps envision crucial, but expected, differences in metabolism between cell types that are similar to previous findings.

**Table 6:** Constraints for each cell type in model simulations for FBA and FVA

Parameter	Diazotroph	Photoautotroph
Carbon Uptake (mmol C (g DW)^-1^ h^-1^)	0.927 (glycogen) [29]	0.927 (CO_2_) [29]
Nitrogen Uptake (mmol N (g DW)^-1^ h^-1^)	Unlimited (N_2_)	Unlimited (NH_4_^+^)
Nitrogenase Flux (mmol (g DW)^-1^ h^-1^)	0.132	0
Maintenance Energy (mmol ATP (g DW)^-1^ h^-1^)	64.3	67.2
***hν*** ^***PSI***^	80	80 − *hν* ^*PSII*^
***hν*** ^***PSII***^	0	80 − *hν* ^*PSI*^
Growth Rate (h^-1^)	0.0146 [29]	0.0146 [29]
Metabolite Output	NH_4_^+^	Glycogen
Objective Function	Maximize biomass	Maximize biomass

Gene and reaction essentiality were also performed using the same constraints and agree well with flux balance analysis (see Additional file 6: Table S6) and gene essentiality in related organisms. Essential genes include carbon fixation, biomass synthesis, and central metabolism (gluconeogenesis and lower glycolysis) for photoautotrophs and nitrogen fixation, biomass synthesis, and carbon oxidative cycles for diazotrophic cells. Most notably, ammonium export is required for biomass production in diazotrophs, predicting that ammonium leakage is necessary for redox/energy balancing in these cells. These findings correlated with previous studies which find that photosynthetic pigments and certain amino acid pathways are essential [42] while some carbon metabolism like the later parts of the TCA cycle are dispensable in cyanobacteria [57]. The model is effective, then, in *in silico* predictions of essential and conserved metabolic motifs.

The flux maps we present here represent only a single possible solution due to the underdetermined system used for optimization. To assess the bounds and possible adaptations of the model, FVA was conducted and visualized for important pathways (Fig. 3). The photoautotrophic cells showed much tighter bounds for central metabolic processes than their diazotrophic counterparts, likely because their metabolites (glycogen) require roughly 64 ATP/mol as opposed to ammonium which requires 8 ATP/mol (16/mol N_2_) plus additional maintenance energy. Consistent with gene essentiality and intuition, Ribulose-1,5-bisphosphate carboxylase/oxygenase (RuBisCO) requires a non-zero flux to maintain optimal behavior. Phosphoglycerate kinase (PGK) – which is in lower glycolysis/gluconeogenesis – is a less intuitive non-zero reaction, but the reaction represents an important step in both optimal carbon oxidation and reduction. For both cell types, D-fructose-1,6-bisphosphate D-glyceraldehyde 3-phosphate lyase (FPAL) and sedoheptulose-7-phosphate: D-glyceraldehyde-3-phosphate (TAGSFE), which correspond to reactions in upper glycolysis and the non-oxidative pentose phosphate pathway, show high variability; TAGSFE can even function reversibly while maintaining optimality. Otherwise, the photoautotrophic cells have zero or near-zero flux for all TCA_Cycle enzymes (acetyl-coA synthase: ACCOASYN, malate dehydrogenase: MDH, isocitrate lyase: ICLY, and bifunctional aconitate hydratase 2/2-methylisocitrate dehydratase: ICIT) because of a lack of electron donors available for carbon oxidation. Glutamine synthase (GLNSYN) displays a small flux for ammonium metabolism and incorporation while PEP carboxylase (PCX) can accommodate flux using its role as an alternative to RuBisCO for single-carbon incorporation. In the diazotrophic cells, pyruvate kinase (PYRK) and glutamine synthase (GLNSYN) exhibit high flux capacities due to their substrates being precursors for many essential metabolites in the cell and the energetic latitude given by the reduced carbon supply, but are irreversible. TCA cycle has intermediate flux capacity because the reactions in it represent best-case processing for carbon oxidation, while the glyoxylate shunt (isocitrate lyase, or ICLY) is used for carbon conservation [53–56]. Diazotrophic metabolism only has one significant deviation: the flux through the nitrogenase enzyme is invariable. These data are similar to findings for other photoautotrophic and diazotrophic organisms [36, 58, 59] and illustrate similar conserved energetic and metabolic behaviors.

### Modeling equilibrium colony composition

Simulations to this point showed the metabolic differences between the cell types, but had not accommodated their function as a community. For this reason, dynamic FBA (dFBA) was used to expand the study to simulate how gene-predicted metabolism causes higher order community behaviors. Previous studies have used dFBA for similar applications, including diauxic growth in *E. coli* and the competition between *Rhodoferax* and *Geobacter* [32, 60, 61], but this study presents the idea of dynamic allocation of cells based on metabolite production to simulate need-based differentiation. Since all cells are defined by the same genome, differentiate based on the needs of the community and are invested in the viability of surrounding cells, there is a third level of interaction within these communities. To model this, diazotrophs and photoautotrophs were treated like separate, symbiotic, interdependent populations that could achieve perfect diffusion of substrate. Then, based on the deficiency of one metabolite, cells would be allocated from the deficiency generating cell (the consumer) in proportion to the shortage to correct it. If there was only metabolite surplus, all excess cells from the diazotroph would be allocated towards photoautotrophic production which was implied to drive biomass growth. This was run with both this and the opposite assumption (that all excess would be allocated to the diazotroph) with identical results, meaning that this assumption was an artifact of the algorithm rather than a pivotal administration. Importantly, this algorithm requires the photoautotrophs to act by generating biomass and enough glycogen for the prior generation plus a predictive amount based on growth rate before the diazotroph reacts and creates ammonium. Therefore, the model can inappropriately fail since metabolites are not being simultaneously synthesized. The model was “seeded” with initial ammonium and glycogen substrates that served to prevent this from affecting the model with a “grace period” of three time steps to allow the model to correct the initial “loan” from the environment. Once the algorithm was developed, it was used to interrogate population efficiency by determining equilibrium compositions of cells, predicting metabolite excretion fluxes, and to predict the effect of changing environments on the cell (especially for conditions that are not possible experimentally). The laboratory imposed constraints used for FBA were converted to relaxed, optimal constraints to reflect the lower growth rates measured in our lab. When optimized independently, assuming replete nutrients for each cell type, both cells grow much faster than in limited, co-dependent conditions (0.0182 h^-1^ for photoautotrophs and 0.0188 h^-1^ for diazotrophs versus 0.0146 h^-1^ for the population). This is logical: when nutrients are limited overall growth is limited. Another observation is that photoautotrophic growth is slower even ideal conditions, reinforcing the hypothesis that biomass generation is limited more by carbon than nitrogen.

To determine equilibrium behavior by the community and how well the model could predict the behaviors we observed, we conducted dFBA using this algorithm over 360 h with several different starting conditions. First, a 50:50 starter culture was used to model both how a colony might achieve equilibrium through cellular differentiation and the resulting equilibrium. In order to achieve efficiency, three phases govern cell response to metabolite deficiencies as illustrated by Fig. 5. First, photoautotrophs are generated to correct for carbon deficiency over the first 120 h (steeper slopes in Fig. 5a and linear slopes in Fig. 5a and d) and without excretion of metabolites (Fig. 5c). Meanwhile, the diazotrophs are tasked with producing enough fixed nitrogen to support the community, but all biomass generation is diverted towards photoautotrophs because the “seed” carbon or nitrogen is rapidly depleted. In the second phase from hours 120 to 250, where the cells are near but not at equilibrium, the cells redistribute more slowly as the community is able to support itself without reaching a deficiency while producing some metabolites (Fig. 5c). Finally, after about 250 h, equilibrium is reached where more photoautotrophs will deplete the environment of fixed nitrogen and fewer will reduce biomass generation. This is also the stage where metabolite production is most rapid, and ammonium and glycogen are exchanged between cells and the environment exponentially Fig. 5. These results are encouraging because they justify our observation that the model converges to an equilibrium regardless of initial composition, and the equilibrium falls within experimental and *in situ* levels.

Convergence toward equilibrium was investigated further by incrementally changing initial biomass ratios and plotting growth rate versus time. Rapid convergence by an inoculum of 15:85 diazotroph:photoautotroph is expected and promotes this as ideal behavior by cellular populations (Fig. 4). The convergence by other inoculums reinforces the idea of an equilibrium composition and shows the inefficiency of other starting populations. Finally, since the model assumes no diffusional limitation and similarly structured cells, this convergence to an equilibrium demonstrates that metabolic interactions play a large role in community regulation of *T. erythraeum*. We repeated this analysis 1000 times to see how the composition of the inoculum affected equilibrium composition of the cells (Fig. 6). In agreement with the single simulation results from above, starting with compositions that were not close to equilibrium resulted in suboptimal growth (for excess diazotrophs) or no growth (for excess photoautotrophs). “Death” (defined as a growth rate of zero for three time steps or more), was strictly contained within the initial phase of growth when the inoculum was 0:100 or 100:0. This is because either nitrogen generation or glycogen generation is too low to support the population before efficient biomass production is achieved and the cells do not have enough exogenous resources to correct this. The equilibrium composition and nitrogen release fraction are both close to observed data: a starter culture closer to the cell composition measured in the ocean (10–20% diazotrophic cells and 80–90% photoautotrophic cells [10, 12–15]) grows nearest the constrained growth rate, while compositions further from that equilibrium result in depressed rates. The full distribution of possible growth rates can be seen in Fig. 7b and illustrates a bimodal distribution. The separation between the two peaks can be explained by the extent of domination by either cell type. When diazotrophs dominate, suboptimal growth occurs; when photoautotrophs dominate, the cells operate optimally or die due to deficient nitrogen production. Figure 7a depicts the range of nitrogen release rates dependent on initial inoculum and display a fairly narrow range. In non-death situations, it is consistent within 25% and 45% of total fixed nitrogen, well within the literature recorded values of 7.7% [62] –52% [22]. Across all growth rates, nitrogen leakage is 29.4% of total fixed nitrogen, but for optimal growth rates ($\frac{\mu }{\mu_{max}}\ge 0.9$), nitrogen leakage is 37.5%. Again, nitrogen leakage appears essential to efficiency in biomass generation as evidenced both by observation and simulation. The repetition of the simulation show that an equilibrium composition similar to observation was invariable and is mediated by metabolism while nitrogen is a required side-effect of this optimal growth.

The model was also used to simulate growth conditions that have not been possible to perform in the laboratory. To date, *T. erythraeum* has not been reported to grow on more reduced nitrogen sources; however, we are able to predict how growth on these other N sources using our model (Fig. 8). With increasingly more reduced nitrogen sources and higher carbon content (N_2_ > NH_4_^+^ > Urea > Glutamate > Glutamine), the growth rate increases from 0.0146 h^-1^ to 0.0262 h^-1^ with the exception of urea, which has the second lowest growth rate (0.0151 h^-1^) due to its requirement of ATP consuming active transport and a byproduct of moderately useful CO_2_. In the Boyle laboratory, *T. erythraeum* only grows on N_2_ or nitrate and this general trend is also seen in other laboratory data. *T. erythraeum*, therefore, lives in a very narrow optimum between two important objectives: carbon and nitrogen fixation. The availability of carbon (or lack thereof) particularly limits growth. In reality, light also has an important role in growth rate because a few centimeters below the surface of the ocean light becomes severely limited. Diffusion, predation, colony shape, and other influential factors can also limit the cell’s ability to grow optimally. Even so, biomass generation predictions correlate well between with laboratory data and predicted equilibrium compositions of cells are close to observed values. In order to more effectively leverage this model for colony modeling more sophisticated methods should be used, however, the model has already enabled genome discovery and investigation into the metabolic regulation of this unique and significant cyanobacterium.

## Conclusions

A genome-scale metabolic network reconstruction was performed for the filamentous diazotrophic cyanobacterium, *T. erythraeum.* This organism has a prominent role in the global nitrogen cycle; it is responsible for 42% of the annual biological nitrogen fixation and secretes between 7.7% [62] and 52% [22] of the nitrogen it fixes into the ocean, providing an important source of bio-available nitrogen to other organisms. This model was then subjected to constraints based modeling, such as FBA, FVA and dFBA to investigate the effect of changing environmental conditions and initial cell composition on equilibrium cell compositions and to predict inter- and intra-cellular fluxes. Our simulations indicate that cells exhibit traditional metabolic diversions to conserve carbon in nitrogen-fixing cells (diazotrophs) and to conserve energy in carbon fixing cells (photoautotrophs). From simulations with unconstrained carbon/nitrogen uptake, it appears that photoautotrophs have a lower optimal growth rate than diazotrophs; however in nature, these conditions would never exist. Diazotrophs rely on photoautotrophs for their required carbon and cannot grow in their absence. The model predicts an optimal growth rate for a trichome made up of 15.4% diaztroph, this agrees well with published reports of 10–20% [10, 12–15]. Thus, the model is capable of accurately predicting the required cellular compositions for optimal growth, implying that the composition of cells within a trichome is largely determined by stoichiometry. The success of this and other correlations between our model and laboratory and *in situ* measurements infers that the hypothesis that metabolites are the main influence for cell differentiation for *T. erythraeum* is correct.

The genome-scale metabolic network reconstruction and subsequent model simulations lay the foundation for further interrogation of the metabolism of *T. erythraeum*. This globally significant diazotroph plays an integral role in the ocean, providing a much needed nitrogen source in a very deplete environment. The modeling techniques we have employed above perform well in terms of predicting the growth of a trichome in an ideal environment; but to fully capture the role of *T. erythraeum* in the ocean, more advanced modeling techniques must be developed. In its native environment, *T. erythraeum* interacts with many other species and therefore multiscale models which can capture not only the interaction of the cells with their environment but interactions between cells (identical and other species) must be developed. These types of multi-scale models may also prove useful in modeling and predicting the effect of rising temperatures and carbon dioxide levels in the atmosphere.

## Methods

### Cell cultivation

*Trichodesmium erythraeum* IMS101 cells were acquired from the Bigelow Laboratory for Ocean Sciences (East Boothbay, ME, USA). They were grown in an New Brunswick (Hamburg, Germany) Innova 44R incubator at 24 °C with 80 μE/m^2^/s with 12h light/12h dark cycles. Cells were grown in artificial seawater YBC-II medium [44] at pH 8.15-8.20. Where specified, KNO_3_ was added to final concentration of 100 μM. All chemicals were obtained from Sigma-Aldrich (St. Louis, MO). Growth rate was monitored by measuring chlorophyll absorbance [63] from 50 mL of culture every two days.

### Biomass composition

Total biomass mass was determined by dry weight analysis, cells were filtered with a Whatman 0.22 *μ*m cellulose-nitrate filter and dried in an oven overnight.

#### Protein quantitation

Total protein was quantified using the Pierce BCA Protein Assay Kit (Waltham, MS, USA). Cyanophycin and phycoerythrin are both protein-based compounds, so to avoid double counting, their mass fractions were subtracted from the total protein content. Proteins were hydrolyzed, derivatized and analyzed on an Agilent 5973 Mass Detector with an Agilent 6890N Network GC System on an HP-5 column to determine the amino acid composition following the method by Antoniewicz et al. [64].

#### Carbohydrate quantitation

Carbohydrates were measured colorimetrically using the anthrone method [65] against glycogen as a standard.

#### Cyanophycin quantitation

Cyanophycin is a primary nitrogen storage molecule for diazotrophs and is polymerized arginine and aspartamine in a 1:1 ratio [66]. Cyanophycin was extracted by disrupting 740 *μ*L of 250 mL cells concentrated to 2 mL via filtration and rinsing with TE buffer with 2.70 mg/mL lysozyme overnight at 37 ºC, centrifuging at 16,100 x G for 5 min, and resuspending the pellet in 1 mL of 0.01 M HCl (in which cyanophycin is soluble) for 2 h. The extraction was repeated on the pellet, the supernatant fractions were combined, and cyanophycin was quantified colorimetrically using the Sakaguchi reaction [67].

#### Phycoerythrin quantitation

Phycoerythrin is a protein-pigment complex that is abundant in *T. erythraeum* and absorbs light for photosynthesis. It is soluble in neutral and slightly basic environments and the supernatant resulting from disruption of 740 *μ*L of cells concentrated from 250 mL to 2 mL via 2.70 mg/mL lysozyme was collected and measured spectrophotometrically at 455 nm, 565 nm, and 592 nm, and quantitated using the equation [*R* − *PE*] = 0.12 × [(*A*_565_ − *A*_592_) − 0.20 × (*A*_455_ − *A*_592_)] [68].

#### Lipid quantitation

Lipids were extracted using the Bligh and Dyer method [69]. Chlorophyll a and phycocyanin are both lipids, so their masses were subtracted from the total lipid to avoid double counting.

Total lipids were assumed to be made up of the four major subclasses: SQDGs, MGDGs, DGDGs, and PGPs [70]. Fatty acid composition was determined via GC/MS to determine the relative carbon length that constituted lipids in total. The carbon length ratios (C_18_:C_16_:C_14_, etc.) was assumed to be the same distribution for every lipid subclass. Otherwise, lipid pathways were consistent with the methods described in the *Cyanothece ATCC51422* model [36] and the relative amounts of lipid subclasses (SQDG:MGDG:DGDG:PGP) were assumed to be consistent with *Cyanothece*.

Fatty acid methyl esters (FAMEs) were analyzed using GC/MS methods previously described for *Synechococcus sp. PCC7002* [71].

#### Chlorophyll A quantitation

The only chlorophyll pigment *T. erythraeum* contains is chlorophyll a, which was extracted using an 80% acetone/20% methanol solvent according to Harris [63]. First, 250 mL cells were filtered using a 0.45 *μ*m glass fiber filter and were then washed with 2 mL solvent to yield a 2 mL green solution. The resulting liquid was centrifuged at 4 °C at 13,100 x G for 10 min and the 1 mL of the supernatant was diluted to 2 mL and assayed spectrophotometrically through measurement at 646.6 nm, 663.6 nm, and 750 nm, and using the equation: [*ChlA*] = [0.01776(*A*_646.6_ − *A*_750_) + 0.00734(*A*_663.6_ − *A*_750_)].

#### Phycocyanin quantitation

2.70 mg.mL lysozyme was used to disrupt 740 *μ*L cells concentrated from 250 mL to 2 mL via filtration overnight and phycocyanin was measured in the resulting medium according to established techniques [72, 73].

#### DNA and RNA quantitation

DNA and RNA were extracted using MoBio UltraClean Microbial Isolation Kits (Carlsbad, CA, USA) with 50 mL cells concentrated to 2 mL, of which 740 *μ*L was disrupted using 2.70 mg/mL lysozyme overnight at 37 °C/proteinase K for 2 h at 55 °C instead of bead disruption. The concentration of DNA and RNA were assessed spectrophotometrically using 1 cm path-length extinction coefficients [74].

### Genome scale metabolic network reconstruction

The genome scale network reconstruction was based on the publically available genome sequence [75] and an automated annotation program [33]. This first draft was manually curated using primary literature [76, 77], enzyme/metabolic databases [34, 35, 78, 79], and comparison to similar organisms [36, 38, 59, 80]. Reactions were considered reversible unless indicated by a database, an annotation in a similar organism, or significant thermodynamic infeasibility to be otherwise. For conserved or metabolically necessary reactions that were not contained in a database, NCBI’s BLAST with an acceptable e-score of 1e-6 [81] algorithm was used and compared to related genera like *Leptolyngbya*, *Anabaena*, *Synechococcus*, and *Cyanothece* to identify unannotated genes. Where these organisms failed to predict genome similarity, proteins displaying the function as the unannotated, or insufficiently annotated, reaction were BLASTed against all available organisms. This resulted in similarities to the organisms in Table 3, and included *Nitrosococcus oceani, Pleurocapsa* sp. PCC 7327, *Zymomonas mobilis* subsp. NRRL B-12526, *Amborella trichopoda, Dehalococcoides mccartyi VS, Nostoc* sp. PCC 7524, and *Candidatus Nitrosophaera gargensis.* Photosynthesis was modeled similarly to other cyanobacterial genome scale reconstructions [36, 38, 59, 80] by converting the complex protein interactions to a series of redox reactions with photons as an initial substrate and subsequent reduction reactions being passed to energy carriers and ATP synthase. Gene-protein-reaction associations were assumed using sequence data in conjunction with existing models and protein structures to predict conditionality of their assignments (e.g., if a reaction required a protein complex, the GPRs were assigned an *and* operator; redundant genes were assigned an *or* operator). These were done with consultation of KEGG [35], BioCyc [34], BRENDA [78], and CyanoBase [79]. Finally, transport reactions included in the model were validated based on proteomic data or diffusion (CO_2_, H_2_O, N_2,_ etc.) [16]. Manual curation efforts built the model out to 1035 reactions; closer inspection of the reactions revealed that several were predicted by the SEED algorithm but had no significant homology to the *T. erythraeum* genome and were non-essential, therefore they were removed. Irreversibility was assumed where databases indicated significant energetic unfavorability (such as in redox reactions) or where other models demonstrated consistent unidirectionality. Reactions were finally elementally and charge balanced using built-in COBRA functionality in conjunction with the PubChem Database [82]. Small molecules with known properties like pyruvate were used to build out into summary biomass reactions and predict summary macromolecule and biomass constitution. Confidence intervals were added to the model to reflect the source of the reported gene linking: fours were assigned to the transport reactions because of their proteomic data, twos were assigned to all reactions with significant sequence similarity to known protein reactions, and ones were assigned to all gap-filling reactions or reactions with insignificant similarity. The model was assessed using built-in COBRA functionality to determine Type III Reactions by closing all exchange reactions and evaluating the flux variability of the internal reactions. This resulted in no variability, meaning that futile cycles were eliminated [83].

### Model simulations

#### Defining constraints

The genome scale metabolic network was used to predict fluxes using FBA [84]. The constraints applied to the model were determined from experimental data or laboratory or *in situ* data found in literature [22, 29]. Two different sets of constraints were used in order to model the different cell types: diazotrophic and photoautotrophic. Diazotrophic involved Photosystem II deactivation [85], nitrogenase activation, and carbon/nitrogen sources available for uptake. Photoautotrophic cells uptake CO_2_ and ammonium while exporting glycogen, diatomic oxygen, and diatomic nitrogen. Available nutrients, trace metals, and ions which are allowed to be used for growth were restricted according to the YBC-II medium formulation. Table 6 details the differences in constraints for each modeled cell type. An upper limit for photon uptake was set to 80 μEinsteins because this represents the total light provided to the cells in the laboratory. Then, biomass related ATP was set to values similar to *Cyanothece* sp. ATCC 51142, another photosynthetic diazotroph [36], at 544 mmol ATP (g DW h)^-1^ and maintenance related ATP was set so that the growth rate matched literature derived physiological data [29]. The model was constrained by the growth rate provided by literature using dFBA to define biomass maintenance energy constraints [29] and then concurrently constraining nitrogenase and carbon dioxide uptake rates from the same study. The maintenance flux for photoautotrophs was determined to be 64.3 mmol (g DW h)^-1^ and 67.2 mmol (g DW h)^-1^ for diazotrophs by matching the growth rate in the dFBA model to the growth rate measured in literature. This is significantly higher than other reported values, but is justified by the lack of substantial light restrictions. It is assumed that light uptake restrictions and maintenance ATP are both contained within this constraint. All other nitrogen and carbon sources except for those specified in Table 6 were set to zero.

#### Problem formulation for FVA and FBA

FBA and FVA were conducted using COBRA toolbox functions [43]. The optimization problem was constructed around the steady-state assumption with objectives of biomass generation subject to different cell type constraints specified by Table 6. Table 7 shows the abbreviations and notations for equations.

**Table 7:** Variables and notations used in simulations

Variables	Identifiers	Sets	Indices
***α***: allocation coefficient	‘: uncorrected value	$\mathcal{C}$: consumers	*γ*: consumer of glycogen/consumer cell in allocation
C: concentration	0: initial	$\mathcal{P}$: producers	c: producer of glycogen
*f:* fraction of cell type	*γ*: consumed metabolite for photoautotroph	$\mathcal{S}$: seawater nutrients	*i*: species
$\widehat{\mu}$: maximum growth rate	c: produced metabolite for photoautotroph		*μ*: consumer of ammonium
***μ***: growth rate	*chIL*: nitrogenase		*n*: producer of ammonium
***ν***: flux	*cons*: consumer		*π*: producer cell in allocation
*N*: number of steps	*δ*: differentiation pool		
***σ***: portion control for allocation	DZ: diazotroph		
*S*: stoichiometric matrix	*f*: final		
*t*: time	*m*: metabolite		
*X*: biomass	*n*: step		
***Y***_***N*** → ***Env***_: fraction of nitrogen released to the environment	PA: photoautotroph		
	*prod*: producer		
	PSI/II: Photosystem I/II		
	T: total		

The cell-specific uptake and export rules as well as the seawater constraints generated a suite of constraints of the form (where DZ means diazotroph, PA means photoautotroph, and M means macroscopic):

The cell-specific uptake and export rules as well as the seawater constraints generated a suite of constraints of the form:

1 $$ -1000\le\ {\boldsymbol{v}}_s^{\boldsymbol{i}}\le\ 0\ \forall s\in \mathcal{S},\ \forall i $$

Diazotroph Cell Bounds:

2 $$ \begin{array}{c}\hfill -1000\le {\boldsymbol{v}}_n^{\boldsymbol{DZ}}\le 0\ \forall n\in \left\{{N}_2, Glycogen,{O}_2\right\}\hfill \\ {}\hfill 0\le {\boldsymbol{v}}_{\mu}^{\boldsymbol{DZ}}\le 1000\ \forall \mu \in \left\{N{H}_4^{+},C{O}_2\right\}\hfill \\ {}\hfill {\boldsymbol{v}}_{PSII}^{\boldsymbol{DZ}}=0\hfill \\ {}\hfill {\boldsymbol{v}}_{chIL}^{\boldsymbol{DZ}}=0.206\hfill \end{array} $$

Phtotoautotroph Cell Bounds:

3 $$ \begin{array}{c}\hfill -1000\le {\boldsymbol{v}}_{N{H}_4^{+}}^{\boldsymbol{PA}}\le 0\hfill \\ {}\hfill 0\le {\boldsymbol{v}}_c^{\mathbf{PA}}\le 1000\ \forall c\in \left\{{N}_2, Glycogen\right\}\hfill \\ {}\hfill 0\le {\boldsymbol{v}}_{PSII}^{\boldsymbol{PA}}\le 1000\hfill \\ {}\hfill \begin{array}{l}{\boldsymbol{\nu}}_{C{O}_2}^{\boldsymbol{PA}}=-0.927\\ {}{\boldsymbol{v}}_{chIL}^{\boldsymbol{PA}}=0\end{array}\hfill \end{array} $$

It should be noted that seawater ingredients are assumed to be replete in this model.

Steady state was assumed to make the model solvable:

4 $$ \boldsymbol{S}\cdot \boldsymbol{v}=0 $$

Essentially, this means that mass is conserved and all inputs exit either through the biomass equation or through export. The second major assumption was that the only outputs would be biomass, glycogen, or ammonium; energy or mass balancing mechanisms, such as through organic acid spilling, were assumed to be negligible because of efficient, exponential growth conditions.

Gene deletions, reaction deletions, and Dead End reaction analysis were run using these constraints. These problem formulations were solved using the COBRA toolbox [43] in conjunction with the Gurobi (Houston, TX, USA) solver in MatLab (Natick, MS, USA).

#### Dynamic FBA (dFBA)

dFBA was executed with the aid of the Dynamic Multispecies Metabolic Modeling (DyMMM) framework as a template [61]. Both this model and the DyMMM model built on traditional dFBA which estimates solutions to the time-dependent equations governing population growth and substrate utilization. This is done using FBA methods to determine interactive fluxes (exchange, biomass, and objective reaction fluxes) from a genome scale. Metabolites were assumed to perfectly diffuse since the relevant study was assumed to center around metabolism and differentiation of cells in response to shortage. The growth rate is also sufficiently slow as to reduce the effects of transport versus diffusion phenomena in ideal, exponential conditions. To model the differentiation between cell types, dFBA allowed a portion of a period’s growth rate to be proportionally allocated to the underproducing cell. If there was no metabolite deficiency in a period, excess growth of all cells was allocated to photoautotrophic cells. Otherwise, the carbon dioxide uptake, nitrogen uptake, and metabolite export rates were constrained only by the requirements of the population (therefore eliminating the strict carbon dioxide and nitrogenase constraints). Only the biomass maintenance energy was constrained to match laboratory growth rates with initial ideal (equilibrium) concentrations of cells at biomass similar to initial biomass measured in laboratory studies. These maintenance fluxes became 64.3 mmol ATP (g DW)^-1^ h^-1^ for the photoautotroph and 67.2 mmol ATP (g DW)^-1^ h^-1^ for the diazotroph.

5 $$ \begin{array}{c}\hfill { \max}_{{\boldsymbol{C}}_{\boldsymbol{Glycogen}}^{\boldsymbol{i}},\boldsymbol{t},{\boldsymbol{\mu}}^{\boldsymbol{i}},{\boldsymbol{v}}_{\boldsymbol{C}{\boldsymbol{O}}_2}^{\boldsymbol{PA}},{\boldsymbol{v}}_{\boldsymbol{m}}^{\boldsymbol{DZ}},{\boldsymbol{f}}_{\boldsymbol{DZ}}}\left({\boldsymbol{\mu}}^{\boldsymbol{PA}}\  and\ {\boldsymbol{v}}_{\boldsymbol{N}{\boldsymbol{H}}_4^{+}}^{\boldsymbol{DZ}}\right)\hfill \\ {}\hfill \boldsymbol{s}.\boldsymbol{t}.{\left[{\boldsymbol{C}}_{Glycogen}^{\boldsymbol{PA}}\right]}_{\boldsymbol{t}}\ge {\left[\left|{\boldsymbol{C}}_{Glycogen}^{\boldsymbol{DZ}}\right|\right]}_{\boldsymbol{t}-1}\ \hfill \\ {}\hfill {\boldsymbol{v}}_{Glycogen}^{\boldsymbol{DZ}}\le \frac{1}{6}\frac{{\boldsymbol{v}}_{C{O}_2}^{\boldsymbol{PA}}}{{\boldsymbol{f}}_{\boldsymbol{DZ}}}\hfill \end{array} $$

For the purpose of this program, the final constraint is estimated (knowing that *μ* and *Δ* ***t*** are non-negative) by:

6 $$ {\left[{\boldsymbol{C}}_{Gly}^{\boldsymbol{PA}}\right]}_{\boldsymbol{t}}=\left({\boldsymbol{\mu}}^{\boldsymbol{DZ}}\varDelta \boldsymbol{t}+1\right){\left[\left|{\boldsymbol{C}}_{Glycogen}^{\boldsymbol{DZ}}\right|\right]}_{\boldsymbol{t}-1}{\left[\frac{{\boldsymbol{X}}_{\boldsymbol{f}}^{\boldsymbol{DZ}}}{{\boldsymbol{X}}_{\boldsymbol{f}}^{\boldsymbol{PA}}}\right]}_{\boldsymbol{t}-1} $$

The dFBA model was built assuming Batch Reactor behavior which has the design equation:

7 $$ \frac{d\boldsymbol{X}}{d\boldsymbol{t}}=\boldsymbol{\mu} \boldsymbol{X} $$

Assuming constant growth rate:

8 $$ {\boldsymbol{X}}_{\boldsymbol{f}}^{\boldsymbol{\hbox{'}}}={\boldsymbol{X}}_0{e}^{\boldsymbol{\mu} \varDelta \boldsymbol{t}} $$

9 $$ \varDelta {\boldsymbol{X}}^{\boldsymbol{\hbox{'}}}=\boldsymbol{X}{\boldsymbol{\hbox{'}}}_{\boldsymbol{f}}-{\boldsymbol{X}}_0 $$

Cells were allocated to a pool of differentiated cells from the preliminary *Δ* ***X*** ', meaning that a cell can only lose as much biomass as it gains in a period. The amount of cells was determined according to the substrate shortage in a period.

10 $$ \begin{array}{c}\hfill {\boldsymbol{C}}_{cons,m}^{\boldsymbol{T}}={\displaystyle \sum_{\gamma }}\left(\varDelta {\boldsymbol{X}}^{\boldsymbol{\gamma}}\cdot {\boldsymbol{v}}_m^{\boldsymbol{\gamma}}\cdot {{\boldsymbol{X}}^{\boldsymbol{\hbox{'}}}}_{\boldsymbol{f}}^{\boldsymbol{\gamma}}\cdot \varDelta \boldsymbol{t}\right)\ \forall \gamma \in {\mathbf{\mathcal{C}}}_m\hfill \\ {}\hfill {\boldsymbol{C}}_{prod,m}^{\boldsymbol{T}}={\displaystyle \sum_{\pi }}\left(\varDelta {\boldsymbol{X}}^{\boldsymbol{\pi}}\cdot {\boldsymbol{v}}_m^{\boldsymbol{\pi}}\cdot {{\boldsymbol{X}}^{\boldsymbol{\hbox{'}}}}_{\boldsymbol{f}}^{\boldsymbol{\pi}}\cdot \varDelta \boldsymbol{t}\right)\forall \pi \in {\mathbf{\mathcal{P}}}_m\hfill \\ {}\hfill {\boldsymbol{\sigma}}^{\boldsymbol{\gamma}}=\frac{{\boldsymbol{C}}_{cons,m}^{\boldsymbol{\gamma}}}{{\boldsymbol{C}}_{cons,m}^{\boldsymbol{T}}}=\frac{\boldsymbol{X}{\boldsymbol{\hbox{'}}}_{\boldsymbol{f}}^{\boldsymbol{\gamma}}\cdot \varDelta \boldsymbol{t}\cdot \left|{\boldsymbol{v}}_m^{\boldsymbol{\gamma}}\right|}{{\displaystyle {\sum}_{\gamma }}\left(\varDelta {\boldsymbol{X}}^{\boldsymbol{\gamma}}\cdot {\boldsymbol{v}}_m^{\boldsymbol{\gamma}}\cdot {{\boldsymbol{X}}^{\boldsymbol{\hbox{'}}}}_{\boldsymbol{f}}^{\boldsymbol{\gamma}}\cdot \varDelta \boldsymbol{t}\right)}\forall \gamma \in {\mathbf{\mathcal{C}}}_m\hfill \\ {}\hfill \begin{array}{l}{\boldsymbol{\sigma}}^{\boldsymbol{\pi}}=\frac{{\boldsymbol{C}}_{cons,m}^{\boldsymbol{\pi}}}{{\boldsymbol{C}}_{cons,m}^{\boldsymbol{T}}}=\frac{\boldsymbol{X}{\boldsymbol{\hbox{'}}}_{\boldsymbol{f}}^{\boldsymbol{\pi}}\cdot \varDelta \boldsymbol{t}\cdot \left|{\boldsymbol{v}}_m^{\boldsymbol{\pi}}\right|}{{\displaystyle {\sum}_{\gamma }}\left(\varDelta {\boldsymbol{X}}^{\boldsymbol{\pi}}\cdot {\boldsymbol{v}}_m^{\boldsymbol{\pi}}\cdot {{\boldsymbol{X}}^{\boldsymbol{\hbox{'}}}}_{\boldsymbol{f}}^{\boldsymbol{\pi}}\cdot \varDelta \boldsymbol{t}\right)}\forall \pi \in {\mathbf{\mathcal{P}}}_m\\ {}\boldsymbol{\alpha} =\frac{{\boldsymbol{S}}_{prod,m}^{\boldsymbol{T}}+{\boldsymbol{S}}_{cons,m}^{\boldsymbol{T}}}{{\boldsymbol{S}}_{cons,m}^{\boldsymbol{T}}}\end{array}\hfill \end{array} $$

The differentiated cell pool, ***X***_***δ***_, was determined by applying the allocation coefficient to the productive cells according to their relative production coefficient:

11 $$ {\boldsymbol{X}}_{\boldsymbol{\delta}}={\displaystyle \sum_{\gamma }}\left(\boldsymbol{\alpha} \cdot {\boldsymbol{\sigma}}^{\boldsymbol{\gamma}}\cdot \varDelta {\boldsymbol{X}}^{\boldsymbol{\hbox{'}}\boldsymbol{\gamma}}\right)\ \forall \gamma \in {\mathbf{\mathcal{C}}}_m $$

12 $$ {\boldsymbol{X}}_{\boldsymbol{f}}^{\boldsymbol{\gamma}}=\varDelta \boldsymbol{X}{\boldsymbol{\hbox{'}}}^{\boldsymbol{\gamma}}+{\boldsymbol{\sigma}}^{\boldsymbol{\gamma}}\cdot {\boldsymbol{X}}_{\boldsymbol{\delta}}+{\boldsymbol{X}}_0^{\boldsymbol{\gamma}}\ \forall \gamma \in {\mathbf{\mathcal{C}}}_m $$

And cells are “dealt” according to those same coefficients for consumers:

13 $$ {\boldsymbol{X}}_{\boldsymbol{f}}^{\boldsymbol{\pi}}=\varDelta {\boldsymbol{X}}^{\boldsymbol{\pi}}+{\boldsymbol{\sigma}}^{\boldsymbol{\pi}}\cdot {\boldsymbol{X}}_{\boldsymbol{\delta}}+{\boldsymbol{X}}_0^{\boldsymbol{\pi}}\ \forall \pi \in {\mathbf{\mathcal{P}}}_m $$

Adjusted values are calculated for final concentrations and, during the next iteration, the new initial values are the final values from the previous iteration. This ends at a defined maximum time (400 h) and cell fractions are calculated as a function of biomass of cell type *i* divided by total biomass. Convergence was assessed using randomized initial compositions, assuming the same total initial biomass amount, and comparing the incremental growth rate, or biomass generation at each time step, as it changed over the time period. Incremental growth rate (*μ*_*t*_), total growth rate (*μ*_*T*_), and fixed nitrogen yield (*Y*_*N* → *Env*_) were calculated by:

14 $$ \begin{array}{c}\hfill {\boldsymbol{\mu}}_{\boldsymbol{t}} = \frac{ \ln \left(\frac{{\displaystyle {\sum}_i}{X}_t^i}{{\displaystyle {\sum}_i}{X}_{t-1}^i}\right)}{1}\hfill \\ {}\hfill {\boldsymbol{\mu}}_{\boldsymbol{T}}=\frac{ \ln \left(\frac{{\displaystyle {\sum}_i}{\boldsymbol{X}}_{\boldsymbol{f}}^{\boldsymbol{i}}}{{\displaystyle {\sum}_i}{\boldsymbol{X}}_0^{\boldsymbol{i}}}\right)}{\varDelta \boldsymbol{t}}\hfill \end{array} $$

15 $$ {\boldsymbol{Y}}_{\boldsymbol{N}\to \boldsymbol{E}\boldsymbol{n}\boldsymbol{v}}=\frac{\varDelta {\boldsymbol{C}}_{\boldsymbol{N}{\boldsymbol{H}}_4^{+}}}{{\boldsymbol{C}}_{N{H}_4^{+}, prod}} $$

Since multiple steady states exist for population composition, growth rate, and substrate release rate, randomized initial cell concentrations (both in terms of composition and initial biomass) were generated using MatLab over 1000 iterations. Also, FBA was run individually on each cell type once more to determine the growth rate of each cell type in replete (independent, unlimited) conditions.

The hypothetical viability of *T. erythraeum* on different nitrogen sources was assessed by “opening” reaction boundaries for exchange reactions corresponding to the nutrient and assuming the nutrient was in excess. Then, the dFBA simulation was run assuming equilibrium starting composition. The growth profiles and growth rates were recorded in the same way as previous simulations. Where proteomics data did not infer transport of a particular compound, a simple, diffusion style transporter was temporarily added to the model.

## Additional files

Additional file 1: Genome scale metabolic reconstruction for *T. erythraeum*. This file contains the full SBML formatted genome scale reconstruction of *Trichodesmium erythraeum* (iTery101) described by this study without constraints. (XML 1720 kb)
Additional file 2: List of reactions and metabolites included in the model. This file contains the more readable format of the Genome scale reconstruction with a catalog of reactions in one tab and of metabolites in the other, supplemented by relevant information for both sets. (XLS 442 kb)
Additional file 3: This file contains the list of dead end reactions generated by the study, not including exchange reactions. This means all reactions which include a metabolite that cannot be otherwise resolved (through reaction to another compound) is included. Dead ends are ignorant to photoautotroph or diazotroph because of the similar genetic background. (XLSX 50 kb)
Additional file 4: MakeTERY function to establish constraints and create separate models. This file contains all relevant constraints for the two different cell types. By default, it also generates the optimal solutions subject to literature constraints as described in the FBA and FVA sections of the paper. (M 2 kb)
Additional file 5: Flux reactions for ideal, exponential growth with fixed biomass/growth rate and objective metabolite production. This file contains the entire simulations data used to generate Fig. 2 and based on the flux balance analysis described by the study. It includes the entire catalog of reactions, the corresponding flux for both diazotroph and photoautotroph, and the reaction formula. (XLSX 58 kb)
Additional file 6: List of effects of gene and reaction deletion identified by *in silico* knockout analysis. This file contains the list of effects from each gene and reaction deletion in separate tabs. Gene/reaction deletions that result in lethality are colored red, those that reduce function are colored yellow, and those that cause no change are white. The single green reaction (deletion of biomass maintenance, EN_ATP) improves gene function. Reactions have associated genes listed alongside and genes have associated reactions listed alongside. There are reports for both diazotrophs and photoautotrophs. Data is reported as ratio (growth rate of knockout divided by “wild type” growth rate), raw knockout growth rate, and change in growth rate calculated using the following equation: $\varDelta \mu =\frac{\mu_{WT}-{\mu}_{mutant}}{\mu_{WT}}$ (XLSX 182 kb)
Additional file 7: List of lower and upper bounds of flux identified by flux variability analysis. Flux Variability for ideal, exponential growth with fixed biomass/growth rate and objective metabolite production with the same bounds as Flux Balance Analysis. Included are lower and upper bounds for acceptable fluxes along with the total range for each reaction. Reactions are organized by the “Euclidean Range”, the square root of the ranges squared, which give an image for the most consistently variable and non-variable reactions between cell types. (XLSX 80 kb)
Additional file 8: Table S1. Average protein composition of *T. erythraeum.* Proteins were hydrolyzed and amino acid concentrations were measured using gas chromatography/mass spectrometry. The molar fraction from ambient air (N_2_) was used for the protein and lipid assembly equations. Starred quantities were derived from a previous study [1] because our method was not able to detect them. **Table S2**. Average lipid composition of *T. erythraeum.* Extracted lipids then analyzed as fatty acid methyl esters on a gas chromatograph/ mass spectrometer. Relative amounts of subclasses of lipids were assumed from previous literature [2]. **Table S3**: Comparison to related genome-scale metabolic network reconstructions. Summary figures for related genome scale reconstructions for relevant photosynthetic organisms. (DOCX 22 kb)

## General

BLAST: Basic local alignment search tool
COBRA: Constraint based reconstruction and analysis
dFBA: Dynamic flux balance analysis
DW: Dry weight
DyMMM: Dynamic multispecies metabolic model
FAME: Fatty acid methyl ester
FBA: Flux balance analysis
FVA: Flux variability analysis
GC/MS: Gas chromatography/mass spectrometry
PSI: Photosystem I
PSII: Photosystem II
RAST: Rapid annotation using subsystems technology
TCA: Tricarboxylic acid

## Enzymes

TAGSFE: sedoheptulose-7-phosphate: D-glyceraldehyde-3-phosphate (E.C. 2.2.1.2)
PCX: PEP carboxylase (E.C. 4.1.1.31)
MDH: Malate dehydrogenase (1.1.1.37)
PYRK: Pyruvate kinase (E.C. 2.7.1.40)
ICLY: Isocitrate lyase (E.C. 4.1.3.1)
ACCOASYN: Acetyl-CoA synthetase (E.C. 6.2.1.1)
PDH: Pyruvate dehydrogenase (E.C. 1.2.4.1)
RuBisCO: D-ribulose-5-phosphate 1-phosphotransferase (E.C. 2.7.1.19)
*chIL*: nitrogenase (E.C. 1.18.6.1)
GLNSYN: L-glutamate: ammonium ligase (E.C. 6.3.1.2)
PGK: 3-phospho-D-glycerate 1-phosphotransferase (E.C. 2.7.2.3)
ATP: ATP synthase (E.C. 3.6.3.14)
FPAL: D-fructose-1,6-bisphosphate D-glyceraldehyde 3-phosphate lyase (E.C. 4.1.2.13)
ICIT: bifunctional aconitate hydratase 2/2-methylisocitrate dehydratase (E.C. 4.2.1.3, E.C. 4.2.1.4)

## Lipid subclasses

DGDG: Digalactosyldiacylglycerol
MGDG: Monogalactosyldiacylglycerol
PG: Phosphatidylglycerol
SQDG: Sulfoquinovosyldiacylglycerol

## Metabolites

6PG: 6-phospho-D-gluconate
6PGDL: 6-phosph-D-glucono-1,5-lactone
AcCoA: Acetyl-CoA
AKG/*α*KG: *α*-ketoglutarate/2-oxoglutarate
ALA: L-alanine
cAMP: cyclic-AMP
CIT: Citrate
CoA: Coenzyme-A
DHAP: Dihydroxyacetone phosphate
E4P: Erythrose-4-phosphate
F6P: Fructose-6-phosphate
FDP: Fructose 1,6-diphosphate
FOR: Formate
FUM: Fumarate
G6P: Glucose-6-phosphate
GAP: Glyceraldehyde 3-phosphate
GLX: Glyoxylate
GLY: Glycine
GLYR: Glycerate
GOL: Glycerol
GP: 3-phosphoglycerate
ICIT: Isocitrate
MAL: Malate
NMP: Nucleotide monophosphate
OAA: Oxaloacetate
PEP: Phospho*enol*pyruvate
PGOL: Phosphoglycolate
PYR: Pyruvate
R5P: Ribose-5-phosphate
Ru5P: Ribulose-5-phosphate
RuBP: Ribulose 1,5-bisphosphate
S17P: Sedoheptulose 1,7-bisphosphate
S7P: Sedoheptulose 7-phosphate
SUCC: Succinate
SUCSAL: Succinic semialdehyde
X5P: Xylulose 5-phosphate
*β*G6P: *β*-glucose-6-phosphate
