# Supplementary material for: The use of genome-scale metabolic network reconstruction to predict fluxes and equilibrium composition of N-fixing versus C-fixing cells in a diazotrophic cyanobacterium, Trichodesmium erythraeum
Source: BMC Syst Biol. 2017 Jan 19;11:4. doi: 10.1186/s12918-016-0383-z (PMC5244712; doi:10.1186/s12918-016-0383-z)
Supplement: Additional file 8: Table S1. — Average protein composition of T. erythraeum. Proteins were hydrolyzed and amino acid concentrations were measured using gas chromatography/mass spectrometry. The molar fraction from ambient air (N2) was used for the protein and lipid assembly equations. Starred quantities were derived from a previous study [1] because our method was not able to detect them. Table S2. Average lipid composition of T. erythraeum. Extracted lipids then analyzed as fatty acid methyl esters on a gas chromatograph/ mass spectrometer. Relative amounts of subclasses of lipids were assumed from previous literature [2]. Table S3: Comparison to related genome-scale metabolic network reconstructions. Summary figures for related genome scale reconstructions for relevant photosynthetic organisms. (DOCX 22 kb) [file 12918_2016_383_MOESM8_ESM.docx]

**SUPPLEMENTAL TABLES**

**Table S1. Average protein composition of *T. erythraeum.*** Proteins were hydrolyzed and amino acid concentrations were measured using gas chromatography/mass spectrometry. The molar fraction from ambient air (N_2_) was used for the protein and lipid assembly equations. Starred quantities were derived from a previous study [1] because our method was not able to detect them.

| Amino Acid | Molar Fraction (mol/mol Protein) × 10^-2^ | |
| --- | --- | --- |
|  | **N_2_** | **NO_3_^-^** |
| Alanine | 4.13 | 4.52 |
| Arginine* | 2.08 | 1.81 |
| Aspartate | 6.60 | 15.8 |
| Asparagine* | 2.40 | 2.09 |
| Cysteine* | 0.453 | 0.393 |
| Glutamine | 55.1 | 46.9 |
| Glutamate | 6.59 | 7.23 |
| Glycine | 4.34 | 4.05 |
| Histidine* | 0.652 | 0.566 |
| Isoleucine | 2.17 | 1.41 |
| Leucine | 2.74 | 3.81 |
| Lysine* | 2.17 | 1.88 |
| Methionine* | 0.915 | 0.795 |
| Phenylalanine | 0.526 | 0.400 |
| Proline | 1.29 | 0.930 |
| Serine | 2.60 | 2.25 |
| Threonine | 2.21 | 2.71 |
| Tryptophan* | 0.648 | 0.563 |
| Tyrosine | 0.894 | 0.669 |
| Valine | 1.48 | 1.21 |

**Table S2. Average lipid composition of *T. erythraeum.*** Extracted lipids then analyzed as fatty acid methyl esters on a gas chromatograph/ mass spectrometer. Relative amounts of subclasses of lipids were assumed from previous literature [2].

| Fatty Acid Methyl Ester | Molar Fraction (mol/mol lipid) ×10^-2^ | |
| --- | --- | --- |
|  | **N_2_** | **NO_3_^-^** |
| 14C | 3.45 | 2.27 |
| 14C:1 | 33.4 | 37.3 |
| 16C | 41.5 | 41.9 |
| 16C:1 | 2.50 | 2.69 |
| 18C | 1.12 | 1.11 |
| 18C:1 | 10.4 | 8.19 |
| 18C:2 | 4.94 | 4.01 |
| 18C:3 | 2.68 | 2.19 |

**Table S3: Comparison to related genome-scale metabolic network reconstructions.** Summary figures for related genome scale reconstructions for relevant photosynthetic organisms.

|  | *Phaeodactylum tricornutum* | *Chlamydomonas reinhardtii* | *Synechocystis* sp. PCC6803 | *Cyanothece* sp. ATCC 51142 | *Trichodesmium erythraeum* |
| --- | --- | --- | --- | --- | --- |
| Genome Size (Mbp) | 27.4 | 112 | 3.57 | 5.46 | 7.75 |
| Subsystems | 90 | 83 | 38 | 51 | 56 |
| Reactions | 1861 | 2190 | 1035 | 625 | 973 |
| Metabolites | 1583 | 1068 | 601 | 587 | 988 |
| Unique Genes | 1025 | 1073 | 677 | 806 | 647 |
| Previous Models | 4 | 5 | 9 | 0 | 0 |
| Reference | [3] | [4] | [5] | [2] | This Study |

**REFERENCES**

1. Vu TT, Hill EA, Kucek LA, Konopka AE, Beliaev AS, Reed JL: **Computational evaluation of Synechococcus sp. PCC 7002 metabolism for chemical production.** *Biotechnology journal* 2013, **8:**619-630.

2. Vu TT, Stolyar SM, Pinchuk GE, Hill EA, Kucek LA, Brown RN, Lipton MS, Osterman A, Fredrickson JK, Konopka AE: **Genome-scale modeling of light-driven reductant partitioning and carbon fluxes in diazotrophic unicellular cyanobacterium Cyanothece sp. ATCC 51142.** *PLoS Comput Biol* 2012, **8:**e1002460.

3. Levering J, Broddrick J, Dupont CL, Peers G, Beeri K, Mayers J, Gallina AA, Allen AE, Palsson BO, Zengler K: **Genome-Scale Model Reveals Metabolic Basis of Biomass Partitioning in a Model Diatom.** *PloS one* 2016, **11:**e0155038.

4. Chang RL, Ghamsari L, Manichaikul A, Hom EF, Balaji S, Fu W, Shen Y, Hao T, Palsson BØ, Salehi‐Ashtiani K: **Metabolic network reconstruction of Chlamydomonas offers insight into light‐driven algal metabolism.** *Molecular systems biology* 2011, **7:**518.

5. Knoop H, Gründel M, Zilliges Y, Lehmann R, Hoffmann S, Lockau W, Steuer R: **Flux Balance Analysis of Cyanobacterial Metabolism: The Metabolic Network of *Synechocystis* sp. PCC 6803.** *PLoS Comput Biol* 2013, **9:**e1003081.
